# Differential Responses of Bovine Monocyte-Derived Macrophages to Infection by *Neospora caninum* Isolates of High and Low Virulence

**DOI:** 10.3389/fimmu.2019.00915

**Published:** 2019-04-30

**Authors:** Marta García-Sánchez, Laura Jiménez-Pelayo, Pilar Horcajo, Javier Regidor-Cerrillo, Einar B. Ólafsson, Amol K. Bhandage, Antonio Barragan, Dirk Werling, Luis Miguel Ortega-Mora, Esther Collantes-Fernández

**Affiliations:** ^1^SALUVET, Animal Health Department, Complutense University of Madrid, Madrid, Spain; ^2^Department of Molecular Biosciences, The Wenner-Gren Institute, Stockholm University, Stockholm, Sweden; ^3^Department of Pathobiology and Population Sciences, Royal Veterinary College, University of London, North Mymms, United Kingdom

**Keywords:** cattle, apicomplexa, host-pathogen, innate immune response, hypermigration, coccidiosis, leukocyte, *Toxoplasma gondii*

## Abstract

*Neospora caninum*, a protozoan parasite closely related to *Toxoplasma gondii*, represents one of the main causes of abortion in cattle. Macrophages (MØs) are mediators of the innate immune response against infection and likely one of the first cells encountered by the parasite during the host infection process. In this study, we investigated *in vitro* how high or low virulent isolates of *N. caninum* (Nc-Spain7 and Nc-Spain1H, respectively) interact with bovine monocyte-derived MØs and the influence of the isolate virulence on the subsequent cellular response. Both isolates actively invaded, survived and replicated in the MØs. However, Nc-Spain7 showed a higher invasion rate and a replication significantly faster, following an exponential growth model, whereas Nc-Spain1H presented a delayed replication and a lower growth rate without an exponential pattern. *N. caninum* infection induced a hypermigratory phenotype in bovine MØs that was characterized by enhanced motility and transmigration *in vitro* and was accompanied by morphological changes and abrogated extracellular matrix degradation. A significantly higher hypermotility was observed with the highly virulent isolate Nc-Spain7. Nc-Spain1H-infected MØs showed elevated reactive oxygen species (ROS) production and IL12p40 expression, which also resulted in increased IFN-γ release by lymphocytes, compared to cells infected with Nc-Spain7. Furthermore, IL-10 was upregulated in MØs infected with both isolates. Infected MØs exhibited lower expression of MHC Class II, CD86, and CD1b molecules than uninfected MØs, with non-significant differences between isolates. This work characterizes for the first time *N. caninum* replication in bovine monocyte-derived MØs and details isolate-dependent differences in host cell responses to the parasite.

## Introduction

*Neospora caninum*, an apicomplexan cyst-forming protozoan, is considered one of the main causes of abortion in cattle worldwide (1, 2). The transmission of *N. caninum* in cattle may occur by ingestion of sporulated oocysts from the environment (horizontal transmission) or, most frequently, transplacental during pregnancy (vertical transmission) by dissemination of tachyzoites from the infected dam to the fetus (1).

Innate defenses triggered by monocytes/macrophages (MØs) are key in the pathogenesis of neosporosis (3). These cells constitute the first line of defense against intracellular infections and play an important role in initiating early innate-immune responses and priming the immune system for the development of adaptive-immune responses (4). It has been demonstrated that *Toxoplasma gondii*, a phylogenetically closely related apicomplexan parasite, has developed strategies to survive, replicate, and disseminate via MØs. Through polarizing the host immune response to its own benefit *T. gondii* is able to persist and establish chronic infections (5). To date, knowledge about *Neospora*-MØ crosstalk is predominantly restricted to mice (4, 6–9) or humans (10), neither of which have been identified as natural hosts for *N. caninum*. Flynn and Marshall (11) published the only *in vitro* study regarding infection of bovine MØ (boMØ) by *N. caninum*, which focused on the description of cytokine production by naïve CD4^+^ T-cells primed by infected MØs.

Another key question in bovine neosporosis is the influence of the parasite isolate on the outcome of infection, an effect that is known for *T. gondii* (12). Several studies have established the rate of invasion and the yield of tachyzoites *in vitro* as phenotypic traits associated with the virulence of *N. caninum* (13). Indeed, there seems to be a clear correlation with the isolate virulence and its efficiency in being transmitted to the growing fetus in mice (14, 15). Furthermore, virulence differences were associated with variation in clinical outcome, infection dynamics and immune responses in pregnant cattle (16, 17). However, the isolate-specific virulence determinants in cattle and specific genetic markers for virulent traits remain unidentified.

*T. gondii* clonal lines have demonstrated differences in virulence based on distinct methods of subverting MØs (18). In the present study, we used a boMØ model of *N. caninum* infection to study the interactions between boMØs and *N. caninum* and identify isolate-specific virulence properties at the cellular level. For this purpose, cells were infected with two isolates of high (Nc-Spain7) or low virulence (Nc-Spain1H), previously characterized *in vitro*, that exhibit marked divergence in transmission and dissemination *in vivo*. Specifically, the highly virulent isolate Nc-Spain7 demonstrates greater infection and proliferation rates compared to the low-virulent isolate Nc-Spain1H *in vitro* and a higher percentage of abortion and vertical transmission (as high as 100%) in a pregnant bovine model (17, 19). In contrast no fetal death occurs in pregnant cattle experimentally infected with the Nc-Spain1H isolate (16).

Thus, we studied initially the ability of both isolates to infect boMØs, to survive and to proliferate in these cells. Thereafter, we studied the impact of *N. caninum* infection on the migratory properties of boMØs. In murine toxoplasmosis, infected dendritic cells (DCs) potentiate parasite dissemination to peripheral organs by a Trojan horse mechanism (20–22). Moreover, *T. gondii* infection shifts DCs into an amoeboid rapid migration mode encompassing cytoskeletal changes with podosome dissolution and reduction of proteolysis of extracellular matrix (23–25). On this basis morphological changes and impact on extracellular matrix degradation of MØs upon *N. caninum* infection were investigated, which were related with induced hypermotility, and enhanced transmigration. Finally, the immunological cell response to infection was characterized by analysis of reactive oxygen species (ROS) production, cytokine expression, induction of IFN-γ release by lymphocytes and changes in cell surface markers.

Our results suggest that differences in *N. caninum* isolates virulence correlate with MØ function, and this represents an important step toward our understanding how this parasite is transmitted across restrictive barriers. In addition, our work shows a direct impact of differences in virulence on the innate and subsequent adaptive immune response generated.

## Materials and Methods

### Ethics Statement

Handling of cows and blood sampling were conducted in accordance with Spanish and EU legislation (Law 32/2007, concerning animals, their exploitation, transportation, experimentation and sacrifice; Royal Decree 53/2013 for the protection of animals employed in research and teaching; Directive 2010/63/UE, related to the protection of animals used for scientific goals). Protocols were approved by the Ethical Committee of the Council of Agriculture, Farming, and Autoctonous Resources of the Principality of Asturias, Spain (permit number PROAE 25/2016) and the Animal Welfare Committee of the Community of Madrid, Spain (permit number PROEX 236/17).

### *In vitro* Generation of Bovine Monocyte-Derived Macrophages

BoMØs were generated as previously described (26, 27) from monocytes isolated from peripheral blood collected from six adult dairy cows testing negative for *N. caninum*, infectious bovine rhinotracheitis virus (IBRV), and bovine viral diarrhea virus (BVDV), both of which are known to induce immunosuppression. Motility, transmigration, cytoskeletal morphology, and gelatin degradation assays were performed using monocytes isolated from bovine peripheral blood purchased from a commercial supplier (Håtunalab AB, Bro, Sweden).

Independent of the source of blood, peripheral blood mononuclear cells (PBMCs) were separated by gradient density centrifugation on Histopaque 1077 (Sigma-Aldrich, USA), and monocytes were isolated by positive selection using mouse anti-human CD14-coupled microbeads (Miltenyi Biotec Ltd., USA) according to the manufacturer's instructions. The identity and purity of monocytes (≥95%) was determined by flow cytometry using a mouse anti-bovine CD14 FITC-labeled antibody (clone CC-G33, Bio-rad Laboratories, USA). Monocytes (CD14^+^ cells) were seeded in 6-well culture plates at a density of 10^6^ cells ml^−1^ in 3 ml of RPMI 1,640 medium (Invitrogen, Life Technologies, UK) supplemented with 10% heat-inactivated fetal calf serum (FCS), 100 IU ml^−1^ of penicillin, 100 μg ml^−1^ of streptomycin, and 50 μM β-mercaptoethanol (Merck Millipore, USA), referred to as complete medium (CM). Cells were incubated at 37°C in the presence of 5% CO_2_ with 100 ng ml^−1^ recombinant bovine (rbo) GM-CSF (Kingfisher Biotech, USA). At day 3, 1 ml of medium from each well was replaced with 1 ml of fresh CM with 100 ng ml^−1^ rboGM-CSF. After 5 days of culture, further evidence of *in vitro* generation of MØs was assessed by light microscopy and flow cytometry based on increased size, increased adherence, cytoplasmic granularity, presence of cell appendages, and expression of surface antigens using antibodies specific for the bovine molecules CD14, MHC Class II, CD80, CD86, CD172a, CD11b, and CD1b) (Supplementary Figure 1).

Prior to parasite infection, MØs were harvested using ice-cold PBS with 2 mM EDTA and soft scraping, reseeded in culture plates at the density of viable cells indicated for each assay and incubated for 24 h to minimize possible cellular stress due to the harvesting procedure. The viability of the cells was checked with a trypan blue exclusion test, and was normally above 95%.

### Parasite Cultures and Macrophage Infection

*N. caninum* isolates of high and low virulence (Nc-Spain7 and Nc-Spain1H, respectively) were obtained from healthy calves infected congenitally (14, 28) and characterized *in vitro* and *in vivo* using murine and bovine models. Virulence differences were based on infection and proliferation rates *in vitro* and percentage of abortion and vertical transmission *in vivo* (14–17). Tachyzoites were routinely maintained in a monolayer culture of the African green monkey cell line (MA-104 clone 8, provided by Hipra Laboratories, SA) as described previously (28). On each passage, the cultures were scraped, passed by 25 G needle, and inoculated onto a new monolayer cell culture. To reduce potential changes in virulence due to prolonged maintenance *in vitro* (29), parasites were used at a passage lower than 15 for all the experiments. Tachyzoites used for boMØ infection were collected from 3 to 3.5 day-growth cultures, when the majority of the parasites (at least 80%) were still in parasitophorous vacuoles, and purified using PD-10 Desalting Columns (G.E. Healthcare, Buckinghamshire, UK) as previously described (13). Viable tachyzoites were counted in a Neubauer chamber via trypan blue exclusion and were inoculated within 1 h of parasite collection.

For cell infection rate (cIR) determination, multiplicities of infection (MOI)s of 3–5 were chosen based on previous *in vitro* studies (30, 31). A MOI of 3 was used in most of the assays to obtain an elevated cIR with a low percentage of multi-infection. For transmigration assay protocol, which requires an increased handling of the MØs, a MOI of 2 was used to ensure cell survival.

The *T. gondii* RFP-expressing Prugniaud line (PRU-RFP, type II) (32) was used as reference line in motility, transmigration, morphology and matrix degradation assays. Tachyzoites were cultured as described for *N. caninum* and collected from 2.5 to 3 day-growth cultures. As a control of phagocytosis and cell response, MØs were inoculated with heat-inactivated (HI) *N. caninum* tachyzoites from Nc-Spain7 and Nc-Spain1H isolates mixed at a ratio 1:1. Purified tachyzoites were killed by incubation at 56°C for 30 min as described previously (33) Lack of viability of tachyzoites was confirmed by trypan blue exclusion and by real-time PCR that measured the expression of *NcTUB*α as previously described (34) in cDNA samples of MA-104 cultures infected with HI tachyzoites for 1 week.

### Cell Infection Rate and Parasite Survival

To study the differential ability of the *N. caninum* isolates to infect and survive in boMØs, cIR defined as the percentage of cells infected with one or multiple tachyzoites using different parasite doses, was determined and compared at 8 and 36 h post-infection (hpi). MØs were cultured in 24-well plates at a density of 2.5 × 10^5^ cells/well and inoculated with live Nc-Spain7 or Nc-Spain1H at a MOI of 3, 4, and 5. In addition, MØs were inoculated with HI tachyzoites at the same MOIs as a control for phagocytosis. Cultures were fixed with 0.05% glutaraldehyde (GA) and 3% paraformaldehyde (PF) and stained using double immunofluorescence staining as described previously (30). Overall number of cells, number of cells containing at least one tachyzoite, and number of cells containing more than one tachyzoite (multi-infected cells) were counted in 10 arbitrarily selected fields using an inverted fluorescence microscope (Nikon Eclipse TE 200, Japan) at a magnification of 200 ×. Counting of events was carried out on images taken with different filters for visualization of nuclei and intracellular and extracellular tachyzoites using a Nikon DSL1 camera; the images were overlaid using Photoshop software (Adobe Systems Incorporated, USA). A mean value of 50 cells was counted in each field by a single operator in order to avoid possible differences due to subjectivity in individual appreciations, and only tachyzoites that retained an unaltered morphology were considered in the count.

To assess the ability of Nc-Spain7 and Nc-Spain 1H to actively invade the host cell, phagocytosis activity of MØs was inhibited by treatment with 10 μM Cytochalasin D (Sigma-Aldrich, Spain) for 30 min, following by washing cells three times with PBS. MØs were cultured, infected, fixed and stained as described above. A MOI of 3 was used for inoculation with Nc-Spain7, Nc-Spain1H, and HI tachyzoites. Parasite invasion was determined in parallel in Cytochalasin D-treated and untreated MØs at 8 hpi.

### Lysosomal Activity of Infected Macrophages

To identify intracellular localization of tachyzoites and their ability to evade degradation, lysosomes were labeled by the addition of LysoTracker Red DND-99 (Thermo Fisher Scientific, USA) into the culture media at a concentration of 75 nM 30 min prior the fixing. Cells were cultured and infected as indicated above using a MOI of 3. At 24 hpi cultures were fixed with 0.05% glutaraldehyde (GA) and 3% paraformaldehyde (PF). Immunofluorescence staining of the parasites is described below.

### Proliferation Assays

Proliferation kinetics of Nc-Spain7 and Nc-Spain1H isolates in MØs were determined by quantifying the number of tachyzoites at specific times (8, 24, 36, 48, 60, and 72 hpi) using quantitative real-time PCR (qPCR). Cells were cultured and infected as indicated above using an MOI of 3. Samples were collected by adding 200 μl of PBS, 180 μl of lysis buffer, and 20 μl of proteinase K (Qiagen, Germany) to each well and were stored at −80°C until DNA extraction. DNA extraction and qPCR were carried out as specified previously (30). Briefly, genomic DNA was extracted using a DNeasy Blood & Tissue Kit (Qiagen, Germany) according to the manufacturer's instructions. Quantification of *N. caninum* DNA was performed via qPCR using a 7,300 Real-Time PCR System (Applied Biosystems, USA). A standard curve of 10^−1^ to 10^4^ tachyzoites was used for the quantification (35). A bovine β-actin standard curve was constructed (from 64 to 0.2 ng DNA/μl) in order to normalize the quantification of the parasites in each sample. The results are expressed as the relationship between the amounts of parasite DNA and cell DNA (*R*^2^ ≥ 0.99; slope values varied from −3.47 to −3.11).

The doubling time (Td), defined as the period of time required for a tachyzoite to duplicate during the exponential multiplication phase, was calculated as previously described (13) by applying non-linear regression analysis and an exponential growth equation using GraphPad Prism (GraphPad Software, USA). The Td for each isolate is presented as the average value obtained from all determinations that revealed a linear regression, *R*^2^ ≥ 0.95 (13). The tachyzoite yield (TY_48h_) was defined as the average number of tachyzoites quantified by qPCR at 48 hpi for each isolate.

In parallel, cells were seeded on coverslips, infected and labeled using a double-immunostaining protocol as described previously (30) to study the proliferation kinetics of both isolates in MØs via microscopy, as a complementary technique to the quantification by qPCR. Three coverslips were photographed for each condition using an inverted fluorescence microscope (Nikon Eclipse TE 200).

### Cytoskeletal Morphology Assay

MØs were seeded on poly-L-lysine (Sigma Aldrich, USA)-coated coverslips at a density of 4 × 10^4^ cells/coverslip and inoculated with Nc-Spain7, Nc-Spain1H, HI tachyzoites, or *T. gondii* (PRU-RFP) tachyzoites (MOI 2). Non-infected MØs were used as the negative control. At 8 hpi, cells were fixed with 0.05% GA and 3% PF and stained as indicated below. The morphology of MØs (100 cells/condition) was analyzed using a Leica DMRB epifluorescence microscope with a 100 × objective and scored on a 0–5 scale according to the following criteria (with non-infected MØs as the reference): (*i)* Podosome structures: present (score 0) vs. reduced (score 1) or absent (score 2); (*ii)* Cell shape: elongated (score 0) vs. rounded (score 1); (*iii)* Filopodia-like extensions: present (score 0) vs. absent (score 1); (i*v)* Presence of membrane veils and/or ruffles: present (score 0) vs. absent (score 1).

### Gelatin Degradation Assay

The *in vitro* gelatinolytic activity of MØs, as a marker for their ability to degrade collagen extracellular matrix, was analyzed by gelatinolysis of Oregon green 488 (OG 488)-conjugated porcine gelatin (Molecular probes, Thermo Fisher Scientific, USA) as described previously (24). Briefly, 2.5 × 10^4^ MØs were inoculated with *N. caninum* Nc-Spain7, Nc-Spain1H tachyzoites or with *T. gondii* (PRU-RFP) tachyzoites (MOI as indicated), deposited on OG-488 gelatin-coated Lab-tek chambers (VWR) and incubated for 24 h in CM. Cells were subsequently fixed with 4% PF and stained with DAPI (Thermo Fisher Scientific, USA). *N. caninum* tachyzoites were stained as indicated below. After fixation and staining, images were generated (160 FOV/ condition, 1 FOV = 0.617 mm^2^) using the 10 × objective of the Zeiss Observer Z1. For each chamber, fluorescence (channels, Ex/Em: DAPI, 360/475; OG-488, 450/525; RFP, 555/585) and phase contrast images covering 0.98/1.6 cm^2^ (61.25% of the total well area) were recorded. Image analysis was automated with the open source software package Cellprofiler (v2.1.1, rev: 9969f42). All images were run through a pipeline designed to define gelatin degradation, non-infected cells and infected cells. Gelatin degradation was defined as loss of signal (gelatin, Oregon green-488). Cells were defined as the Euclidian center of a signal (nuclei) + 15 μm radius. Infected cells were defined as the presence of signal (*N. caninum*: Alexa 594 or *T. gondii*: RFP) overlapping with a Euclidian center of signal (nuclei) + 15 μm radius. Degradation was then ascribed to each cell population by overlaying non-infected and infected cells with degradation. Co-ascribed degradation was omitted.

### Motility Assay

The motility assay was conducted as previously described (23). Briefly, MØs were seeded in a 96-well plate at a density of 2 × 10^4^ cells/well and inoculated with *N. caninum* Nc-Spain7, Nc-Spain1H, or HI tachyzoites (MOI 3). In addition, MØs inoculated with *T. gondii* (PRU-RFP, MOI 3) tachyzoites were used for motility comparisons, and non-infected MØs served as the negative control. Prior to infection, *N. caninum* tachyzoites were incubated for 15 min with CMTMR dye (2.5 μM, Thermo Fisher Scientific, USA). Bovine collagen I (0.75 mg ml^−1^, Thermo Fisher Scientific, USA) was added at 4 hpi, and cells were imaged every min for 60 min with a 10 × objective (Zeiss Observer Z1, Zen 2 Blue v.4.0.3). The infection rate was determined, and the absence of differences between groups of inoculated MØs was confirmed (data not shown). Motility data were obtained from 50 cells/condition using ImageJ (Manual Tracking and Chemotaxis and Migration tool plugins).

### Transmigration Assay

Transmigration assays were performed as previously described (20), with minimal modifications. Briefly, MØs were seeded at a density of 1 × 10^6^ cells/well and inoculated with Nc-Spain7, Nc-Spain1H, or HI tachyzoites at a MOI of 2. *T. gondii*-infected MØs (PRU-RFP) were also included in the study as above, and non-infected MØs were used as the negative control. At 6 hpi, MØs were recovered by soft scraping, and 3 × 10^5^ cells/condition were transferred to transwell filters (8 μm pore size; BD biosciences, San José, CA, USA) and incubated for 16 h at 37°C with 5% CO_2._ In addition, 2 × 10^4^ cells/condition were seeded on coverslips, fixed and stained as indicated below, and the infection rate was determined, confirming the absence of differences between groups of inoculated MØs (data not shown). Migrated MØs were recovered by trypsinization and quantified using a Neubauer chamber.

### Analysis of ROS Generation

Analysis of intracellular ROS generation by infected MØs was performed via flow cytometry. MØs were seeded in 6-well plates at a final density of 1.5 × 10^6^ cells/well and incubated in CM for 1 or 24 h post inoculation with Nc-Spain7, Nc-Spain1H or HI tachyzoites (MOI 3). MØs inoculated with H_2_O_2_ (2 μM) were used as the positive control and non-infected MØs as the negative control. Cells were then harvested and stained with 5 μM CellROX Green Reagent (Thermo Fisher Scientific, USA) for 30 min at 37°C, and the fluorescence of viable cells, gated for propidium iodide (5 μg ml^−1^) staining, was assessed in a FACS Calibur cell Analyzer (BD Bioscience, USA).

### Analysis of Bovine IL-10 and IL-12 Cytokine Expression

The mRNA expression levels of IL-10 and IL-12p40 were determined by quantitative reverse-transcription real-time PCR (RT-qPCR). MØs were seeded at a density of 10^6^ cells ml^−1^ in 6-well culture plates and inoculated with Nc-Spain7, Nc-Spain1H, and HI tachyzoites at MOI 3 or with LPS (100 ng ml^−1^) as the control for MØ activation for 8 h and then resuspended in 300 μl of RNAlater (Qiagen, Germany). Non-infected MØs were used as the negative control. Samples were recovered 8 h later by scraping and centrifugation at 1,350 × g for 15 min at 4°C. The obtained pellets were resuspended in 300 μl of RNAlater (Qiagen, Germany) and stored at −80°C until RNA extraction. Analysis was performed on three replicates obtained from three independent experiments. RNA was extracted using the commercial Maxwell 16 LEV simply RNA Purification Kit (Promega, USA) following the manufacturer's recommendations. Integrity was checked by 1% agarose gel and RNA concentrations were determined using a spectrophotometer (Nanophotomer, Implen GmbH, Germany). cDNA was obtained by reverse transcription using a master mix SuperScript VILO cDNA Synthesis Kit (Invitrogen, UK), and IL-10 and IL-12p40 mRNA expression was determined using the primers and conditions previously described (17). Genes were considered to be differentially expressed when they presented a fold change (FC) ≥2 and *p* < 0.05.

### Surface Marker Expression Analyses of Immune Cell Subsets by Flow Cytometry

Surface expression of CD14, MHC Class II, CD80, CD86, CD172a, CD11b, and CD1b by boMØs after exposure to *N. caninum* was determined by flow cytometry. To do so, MØs were seeded in 24-well plates at a density of 3 × 10^5^ cells/well and inoculated with Nc-Spain7, Nc-Spain1H, and HI tachyzoites (MOI 3) or LPS (100 ng ml^−1^) for 4 h as the control for MØ activation. After 4 h, cells were detached from the culture plates by incubation with cold PBS with 2 mM EDTA at 4°C for 10 min and soft scraping. Cultures were recovered in 30 ml centrifuge tubes and centrifuged at 1,200 rpm for 5 min at 4°C. After centrifugation, cells were resuspended in cold PBS at a density of 2 × 10^5^ cell/100 μl. A volume of 100 μl of cells per well was pelleted in a V-bottom 96-well plate and centrifuged at 1,300 rpm 4°C for 3 min. PBS was discarded and cells were incubated with 50 μl of diluted antibody (1:100) or 50 μl of PBS for 30 min on ice and protected from light. After the incubation, samples were washed with PBS before adding the fixative BD Cellfix (BD Bioscience, USA).

The negative fraction (CD14^−^ cells) obtained during positive selection of monocytes (CD14^+^ cells) and used for determination of IFN-γ secretion assay was characterized following the protocol described for surface marker analysis of MØs. The percentage of CD4 T cells (CD4^+^), CD8 T cells (CD8^+^), Natural Killer (NKp46, CD335^+^), gamma delta T cells (WC1^+^), and B cells (CD21^+^) in the population was determined (Supplementary Figure 2).

The percentage of positive cells and mean fluorescence intensity (MFI) was measured for each marker using a Becton Dickinson FACSCalibur cytometer (BD Bioscience, USA). The data were analyzed using FlowJo software (FlowJo, LLC, USA). The list of antibodies used to analyze subsets is given in Supplementary Table 1.

### Determination of IFN-γ Secretion by Lymphocytes

IFN-γ secretion was measured in supernatants of lymphocytes incubated with MØs. Lymphocytes used in the assay (CD14^−^ cells) were obtained as the flow through after magnetic isolation of CD14^+^ cells. This cell population, characterized by flow cytometry as indicated below, contained CD4 T cells (26.53% ± 1.45), CD8 T cells (34.05% ± 1.07), B cells (18.06% ± 0.77) Natural Killer (8.46% ± 0.23), and γδ T cells (18.26% ± 1.14). MØs were seeded in 24-well plates at a density of 2.5 × 10^5^ cells/well and inoculated with Nc-Spain7, Nc-Spain1H or HI tachyzoites at MOI 3 or with LPS (100 ng ml^−1^). Twenty-four hours later, lymphocytes were added at a density of 12.5 × 10^5^ cells/well. To verify that IFN-γ was secreted in our samples exclusively by lymphocytes upon induction by infected MØs, MØs inoculated at the conditions previously described without lymphocyte addition and lymphocytes without MØs were included in the experiment as controls. Supernatants were recovered 48 and 72 hpi and bovine IFN-γ concentrations were determined using a bovine IFN-γ ELISA development kit (Mabtech AB, Sweden), following the manufacturer's recommendations. The color reaction was developed by the addition of 3,3′,5,5′-tetramethylbenzidine substrate (TMB, Sigma-Aldrich, Spain) and incubated for 5–10 min in the dark. Reactions were stopped by adding 2N H_2_SO_4_. Then, plates were read at 450 nm. The cytokine concentrations were calculated by interpolation from a standard curve generated with recombinant cytokines provided by the kit.

### Immunofluorescence Staining of Macrophages and *N. caninum* Tachyzoites

For the gelatin degradation assay, a single immunofluorescence staining of tachyzoites was carried out as described previously (30). After permeabilization with Triton X-100 (Thermo Fisher Scientific, USA), parasites were stained using hyperimmune rabbit antiserum directed against *N. caninum* tachyzoites (1:1,000) as the primary antibody (36) and goat anti-rabbit IgG conjugated to Alexa Fluor-594 (Thermo Fisher Scientific, USA) (1:1,000) as the secondary antibody.

For cIR determination, intracellular proliferation and cytoskeletal morphology assays, a double immunofluorescence staining of tachyzoites was carried out as described previously (30). Parasites were stained using hyperimmune rabbit antiserum directed against *N. caninum* tachyzoites (1:1,000) as the primary antibody. For the double immunostaining, when F-actin filaments were stained (intracellular proliferation assay) with Alexa Fluor-594 Phalloidin (Thermo Fisher Scientific, USA) or non-stained (cIR determination), a 1:1,000 dilution of goat anti-rabbit IgG conjugated to Alexa Fluor-488 (Thermo Fisher Scientific, USA) was added before permeabilization with Triton X-100 to stain extracellular tachyzoites. After permeabilization, a 1:1,000 dilution of goat anti-rabbit IgG conjugated to Alexa Fluor 594 was used to stain both extra- and intracellular tachyzoites. When Alexa Fluor-488 Phalloidin was used for the staining of F-actin filaments (morphology assay), donkey anti-rabbit Alexa Fluor 350 (Thermo Fisher Scientific, USA) was added to stain extracellular tachyzoites, and goat anti-rabbit IgG conjugated to Alexa Fluor 594 (Thermo Fisher Scientific, USA) was used to stain both extra- and intracellular tachyzoites. The nuclei were stained by washing the cells with a solution of 1:5,000 DAPI (Thermo Fisher Scientific, USA) in PBS, and the coverslips were embedded in Fluoroprep (BioMerieux, France).

For the study of the lysosomal activity of infected MØs, cells were permeabilized with 0.5% saponin (Fluka BioChemika, Spain). Parasites were stained using hyperimmune rabbit antiserum directed against *N. caninum* tachyzoites (1:1,000) as the primary antibody and goat anti-rabbit IgG conjugated to Alexa Fluor-488 (Thermo Fisher Scientific, USA) (1:1,000) as the secondary antibody.

### Statistical Analysis

After testing the samples for normal distribution, differences in cIR, proliferation, and cytokine expression were analyzed using a non-parametric Kruskal-Wallis test followed by Dunn's *post-hoc* test for comparisons between groups. Mann–Whitney *U*-test was used for pairwise comparisons. ROS generation, IFN-γ secretion, surface marker detection, motility, transmigration, cytoskeletal morphology, and gelatin degradation assay data were analyzed via parametric one-way ANOVA, followed by Tukey's *post-hoc* test for multiple comparisons and Student's *t*-test for pairwise comparisons. Dunnett's test was used to compare all the groups to the non-infected group. Statistical significance was established at *p* < 0.05.GraphPad Prism 7 v.7.04 (San Diego, CA, USA) software was used to perform all statistical analyses and graphical illustrations.

## Results

### The Highly Virulent Isolate Nc-Spain7 Replicates Significantly Faster in Bovine Macrophages

**Figure 1:**
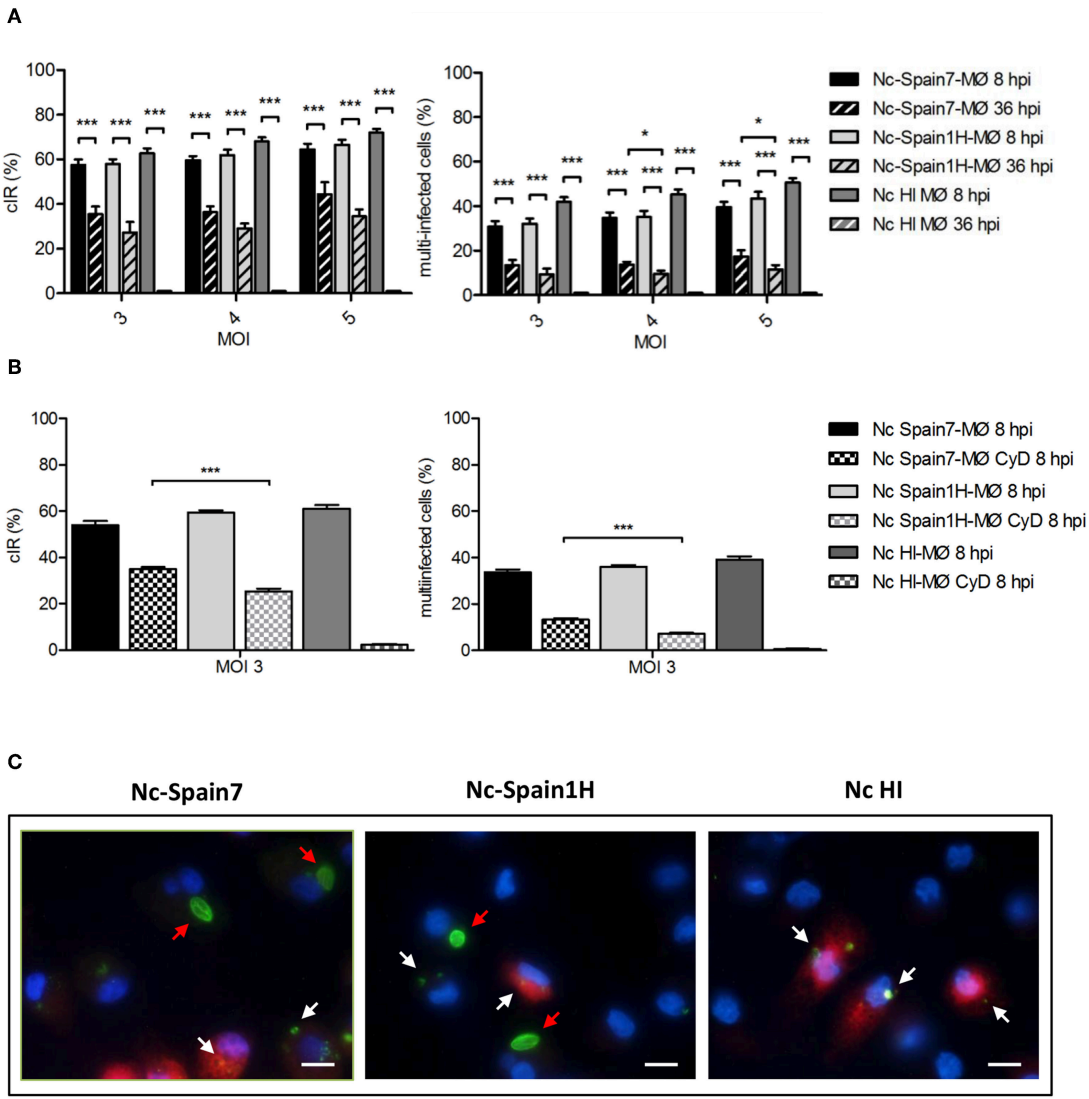
Active invasion and parasite survival of Nc-Spain7 and Nc-Spain1H isolates in bovine MØs. **(A)** Graphs represent the cell infection rates at 8 and 36 hpi as the percentage of infected cells and multi-infected cells in bovine MØs for both isolates. Each column and error bar represents the mean value and SEM of three replicates from three independent experiments using MOIs of 3, 4, and 5. The total number of cells, the number of infected cells (with intact tachyzoites) and the number of cells with multi-infection were determined by double immunofluorescence staining followed by counting using an inverted fluorescence microscope. Degraded tachyzoites were found inside MØs inoculated with HI tachyzoites at 36 hpi. Significant differences are indicated (*p < 0.05; ***p < 0.001). **(B)** Graphs represent the cell infection rates at 8 hpi as the percentage of infected cells and multi-infected cells in untreated and Cytochalasin D treated MØs (MØ CyD) for both isolates. Each column and error bar represents the mean value and SEM of three replicates from three independent experiments using a MOI of 3. Significant differences are indicated (***p < 0.001). **(C)** Representative images (at 1,000×) show the proliferation of Nc-Spain7 and Nc-Spain1H isolates in bovine MØs (red arrows) and the degradation of phagocyted tachyzoites in phagolysosomes (white arrows). Lysosomes stained with LysoTracker Red DND-99 are presented in red, nuclei in blue and parasites in green. Scale bar is 10 μm.

In an initial experiment, we compared the ability of *N. caninum* isolates of different virulence to complete their life cycle in boMØs. First, we evaluated the capacity of the parasite to infect and survive in boMØs. At 8 hpi, MØs internalized tachyzoites with the same efficiency for all assayed conditions. The cIR and the percentage of multi-infected cells did not increase significantly with increasing the MOI, and non-significant differences were found between isolates or between live vs. HI tachyzoites (Figure 1A, *p* > 0.05). To study the ability of both isolates to survive in MØs, cIR and the percentage of multi-infected cells was also evaluated at 36 hpi. The time point of 36 hpi was chosen to permit degradation of dead tachyzoites in order to count only live tachyzoites. Discerning between live and dead tachyzoites was also facilitated by the higher multiplication of the parasites at that time-point. Tachyzoites with unaltered morphology (regarding size, shape, and keeping a smooth surface in the absence of visible defects on its membrane) were counted in MØs infected with Nc-Spain7 and Nc-Spain1H isolates. However, only degraded tachyzoites were observed in MØs inoculated with HI tachyzoites at 36 hpi. A statistically significant reduction in cIR (from 61.34 ± 12.4 to 34.94 ± 14.35; *p* < 0.001) and the percentage of multi-infected cells (from 35.91 ± 15.08 to 12.50 ± 8.74; *p* < 0.001) was shown for both isolates at 36 hpi compared with 8 hpi at all MOIs assayed (Figure 1A). Additionally, when comparing both isolates at 36 hpi, the highly virulent isolate Nc-Spain7 showed a higher multi-infected cell number (*p* < 0.05 for MOI 4 and 5) than the low virulence isolate Nc-Spain1H.

As the presence of intracellular tachyzoites can be either based on active invasion or actin-filament dependent phagocytosis, we next assessed these possibilities by disrupting the actin skeleton using Cytochalasin D. After inhibition of phagocytosis, Nc-Spain7 showed a higher cIR and percentage of multi-infected cells (34.96 ± 5.89 and 13.22 ± 4.89, respectively; *p* < 0.001) compared to Nc-Spain1H (25.45 ± 6.80 and 7.09 ± 3.33) at 8 hpi (MOI 3). Cytochalasin D-treated MØs inoculated with HI tachyzoites showed a cIR of 2.24 ± 2.68, with a percentage of multi-infected cells of 0.44 ± 0.75. No differences were observed between cIR in Cytochalasin D-treated MØs at 8 hpi (Figure 1B) vs. cIR at 36 hpi (Figure 1A).

Having established that tachyzoites of both isolates are able to actively invade boMØs, we next assessed whether these would thus also evade lysosomal degradation at 24 hpi. As expected based on the results obtained with Cytochalasin D, MØs exposed to HI tachyzoites showed clear co-localization of LysoTracker, and degraded tachyzoites. In contrast, this was absent for tachyzoites of both strains in replication, indicating no fusion of the parasitophorous vacuole with lysosomal compartments (Figure 1C). These results show that those tachyzoites internalized by phagocytosis suffer the macrophage driven degradation effects.

**Figure 2:**
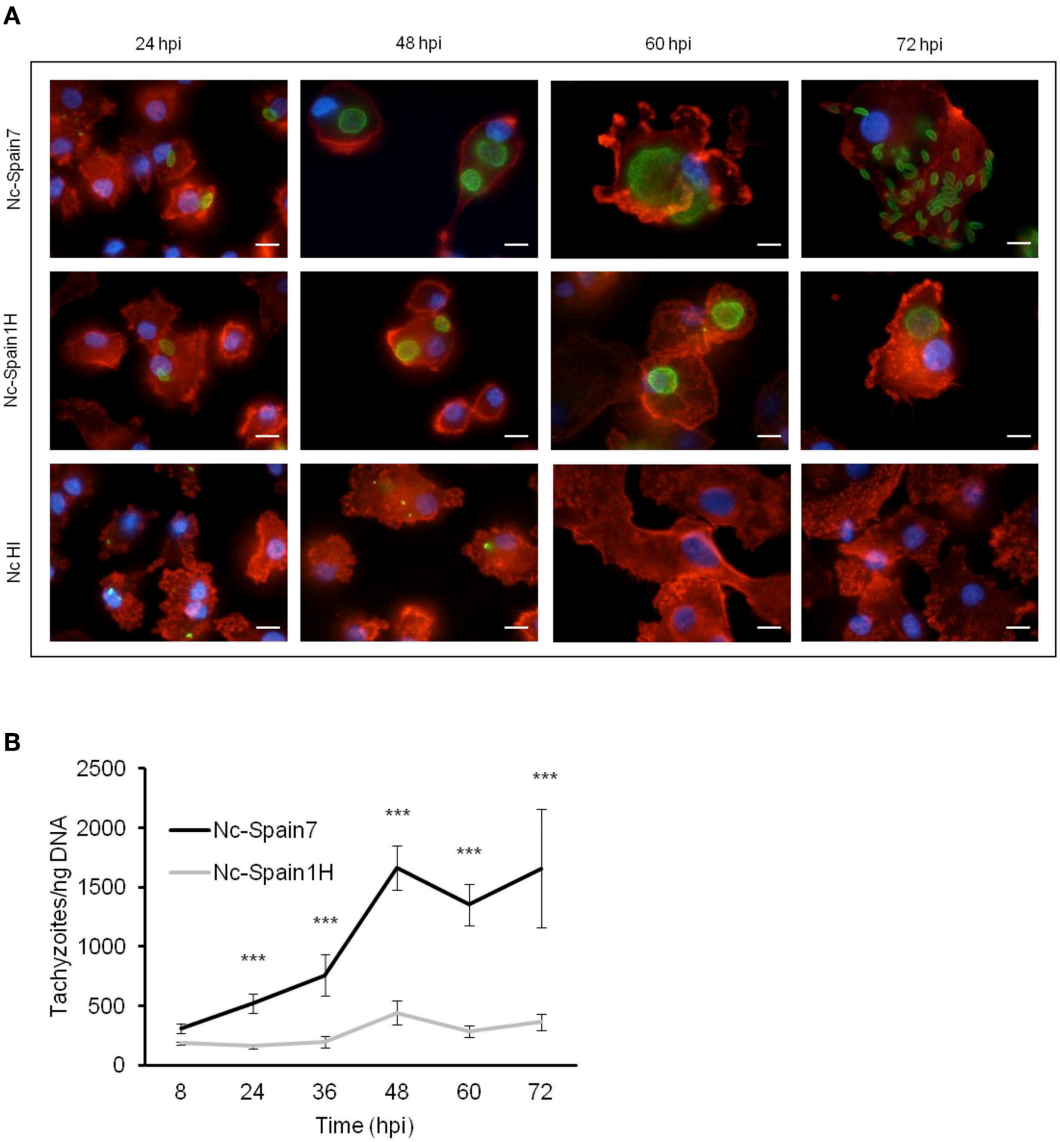
Proliferation of Nc-Spain7 and Nc-Spain1H isolates in bovine MØs. **(A)** Representative images (at 1,000×) show the proliferation kinetics in MØs over time of Nc-Spain7 and Nc-Spain1H isolates and the degradation of HI tachyzoites in bovine MØs cultures. F-actin is presented in red, nuclei in blue and parasites in green. Scale bar is 10 μm. **(B)** The graph represents the average number of tachyzoites for each time point assayed. The growth of Nc-Spain1H in bovine MØs did not fit the exponential growth equation. Error bars indicate the SD. Significant differences are indicated (***p < 0.001).

As a second marker to identify potential differences, we assayed the ability of both *N. caninum* isolates to proliferate in boMØs *in vitro*. Microscopic examination of cultures infected at a MOI of 3 and fixed at different times post-infection showed that Nc-Spain7 and Nc-Spain1H tachyzoites had already begun to multiply at 24 hpi. Parasitophorous vacuoles of Nc-Spain7 were bigger than Nc-Spain1H vacuoles from 48 hpi onwards. For Nc-Spain7, rupture of the host cells and egression of the tachyzoites began to be visualized from 48 hpi and was complete before 72 hpi, at which time point, invasion of new cells by the egressed tachyzoites was observed. For Nc-Spain1H egression did not begin until 60 hpi. At 72 hpi egression is not completed, since intact vacuoles were still visualized (Figure 2A). Proliferation kinetics over time assessed by qPCR are presented in Figure 2B. From 24 hpi onwards, the average number of tachyzoites was higher for Nc-Spain7 for each time point assayed (*p* < 0.001). The Td value was analyzed to determine the length of the cell cycle for both isolates. Nc-Spain7 showed an exponential growth with an average Td value of 13.15 ± 3.64. Nc-Spain1H failed to produce an exponential pattern growth, and thus, their Td value could not be calculated. The TY_48h_ was also assessed to determine the number of tachyzoites produced during the same intracellular period after invasion, prior to complete tachyzoite egress from cell cultures (Figure 2B). The TY_48h_ value was four times higher in Nc-Spain7-infected cultures (1,663 ± 185) than in Nc-Spain1H-infected cultures (442 ± 99; *p* < 0.001).

Altogether, these findings demonstrated that *N. caninum* was able to infect, survive, and complete the parasite lytic cycle in boMØs. Remarkably, a higher active invasion rate and proliferation over time was observed for the highly virulent isolate Nc-Spain7. We therefore analyzed in the next steps whether this increased rate was due to a more severe impact of this isolate in immune function of MØs.

### Active Invasion by *N. caninum* Tachyzoites Leads to Isolate-Specific Morphological Changes in Bovine Macrophages

**Figure 3:**
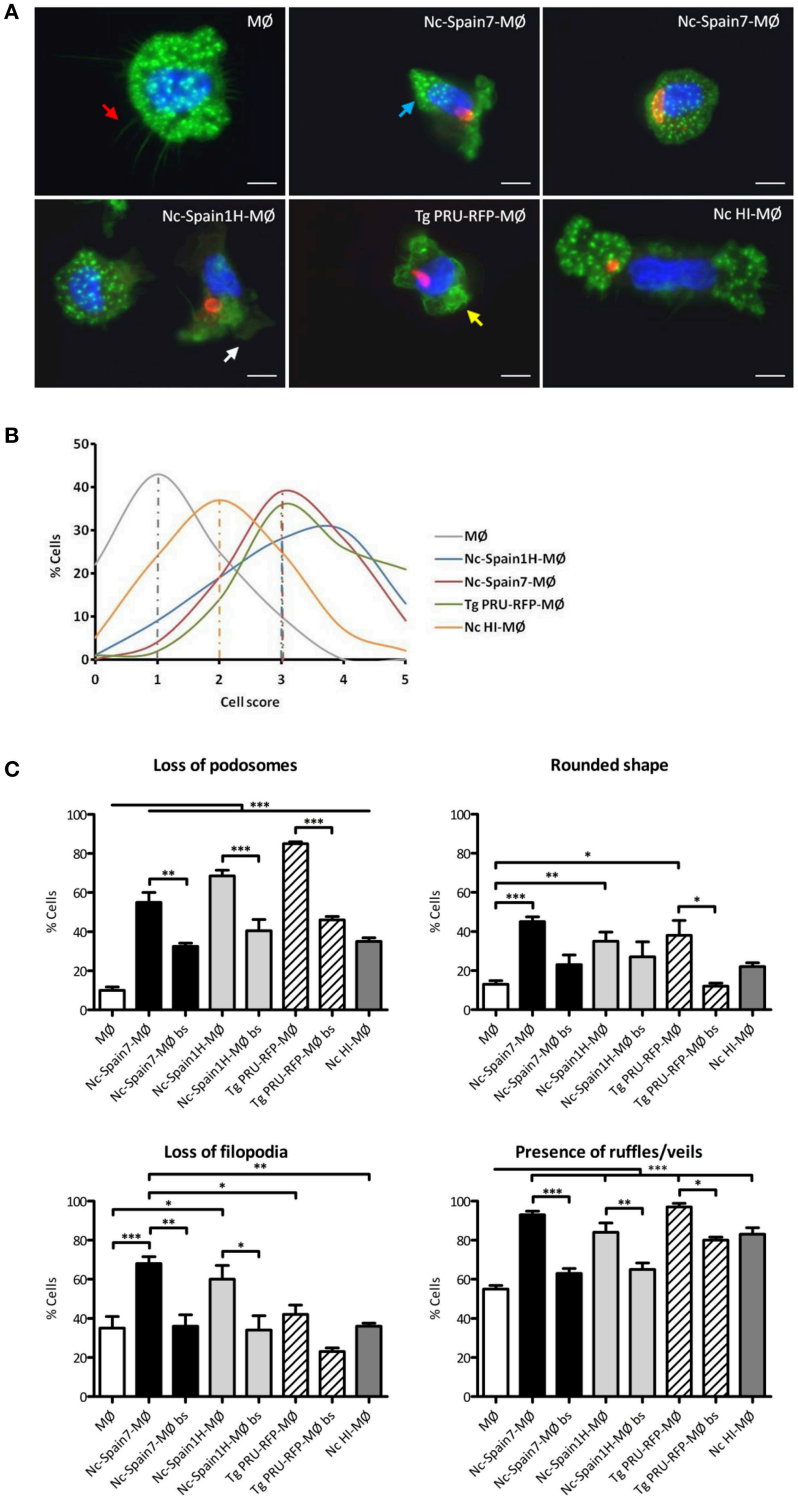
Morphological changes in bovine MØs induced by *N. caninum* and *T. gondii* active invasion. **(A)** Representative micrographs of non-infected MØs (MØ) and MØs challenged for 8 h with *N. caninum* Nc-Spain7 (Nc-Spain7-MØ), Nc-Spain1H (Nc-Spain1H-MØ), heat-inactivated tachyzoites (Nc HI-MØ), or *T. gondii* PRU-RFP (Tg PRU-RFP-MØ). F-actin is presented in green, nuclei in blue and parasites in red. Cell structures studied are pointed with arrows: podosomes (blue), filopodia-like extensions (red), ruffles (yellow), and veils (white). Scale bar is 10 μm. **(B)** The distribution of the total scores obtained are presented as the percentage relative to the total population of MØs, Nc-Spain7-MØs, Nc-Spain1H-MØs, Tg PRU-RFP-MØs, and Nc HI-MØs. Cells were graded according to the following morphological criteria: (*i*) Podosome structures: present (score 0) vs. reduced (score 1) or absent (score 2); (*ii*) Cell shape: elongated (score 0) vs. rounded (score 1); (*iii*) Filopodia-like extensions: present (score 0) vs. absent (score 1); *iv*) Membrane veils and/or ruffles: present (score 0) vs. absent (score 1). **(C)** Bar graph indicates the percentage of cells that exhibited podosome loss, a rounded shape, filopodia-like extension loss and the presence of ruffles or veils, relative to the total cell population of unchallenged, infected and non-infected by-stander MØs (bs). A total of 100 cells were analyzed for each condition on four coverslips from two independent experiments. Significant differences are indicated (*p < 0.05; **p < 0.01; ***p < 0.001).

Upon infection of human or murine DCs, *T. gondii* rapidly induces cytoskeletal changes which have been linked to the migratory activation of the parasitized cell (23, 37). To analyze the cytoskeletal morphology of boMØs challenged with *N. caninum* and *T. gondii*, F-actin filaments were stained and MØs were scored according to set criteria, detailed under *Materials and Methods*. A dramatic impact on cytoskeletal parameters was observed upon challenge with parasites (Figure 3A), with significant differences in total scores between *T. gondii-* or *N. caninum*-infected MØs (median score = 3) and unchallenged MØs (median score = 1) (*p* < 0.001). BoMØs challenged with HI tachyzoites and by-stander boMØs (non-infected cells in challenged cell cultures) exhibited a median score of 2 (Figure 3B). Significant score differences were observed between groups when the morphology parameters were studied separately (Figure 3C). Infection with *T. gondii* resulted in cytoskeletal remodeling characterized by an increase in the percentage of cells that exhibited a rounded shape (*p* < 0.05), total absence of podosomes (*p* < 0.001), maintenance of filopodia-like extensions and a higher presence of ruffles, and/or veils (*p* < 0.001). For Nc-Spain7, the most frequently observed phenotype was partial loss of podosomes (*p* < 0.001), loss of filopodia-like extensions (*p* < 0.01) and a higher presence of ruffles and/or veils (*p* < 0.001). Significant differences were also observed in the percentage of cells that exhibited a rounded shape (*p* < 0.01). The morphology of Nc-Spain1H-infected MØs was chiefly characterized by total absence of podosomes (*p* < 0.001), an elongated or dysmorphic shape and a higher frequency of ruffles and/or veils (*p* < 0.001). HI and by-stander cells exhibited an elongated shape, a partial or null loss of podosomes, presence of filopodia, and an increment of ruffles/veils (*p* < 0.001).

Overall, we conclude that the intracellular presence of live *N. caninum* induced cytoskeletal remodeling in boMØs consistent with cellular activation. The morphological impact was similar to that observed for *T. gondii* but significantly different from the morphological changes induced by HI *N. caninum* or those observed in by-stander boMØs.

### *N. caninum* Infection Reduces Pericellular Proteolysis of Extracellular Matrix by Bovine Macrophages

**Figure 4:**
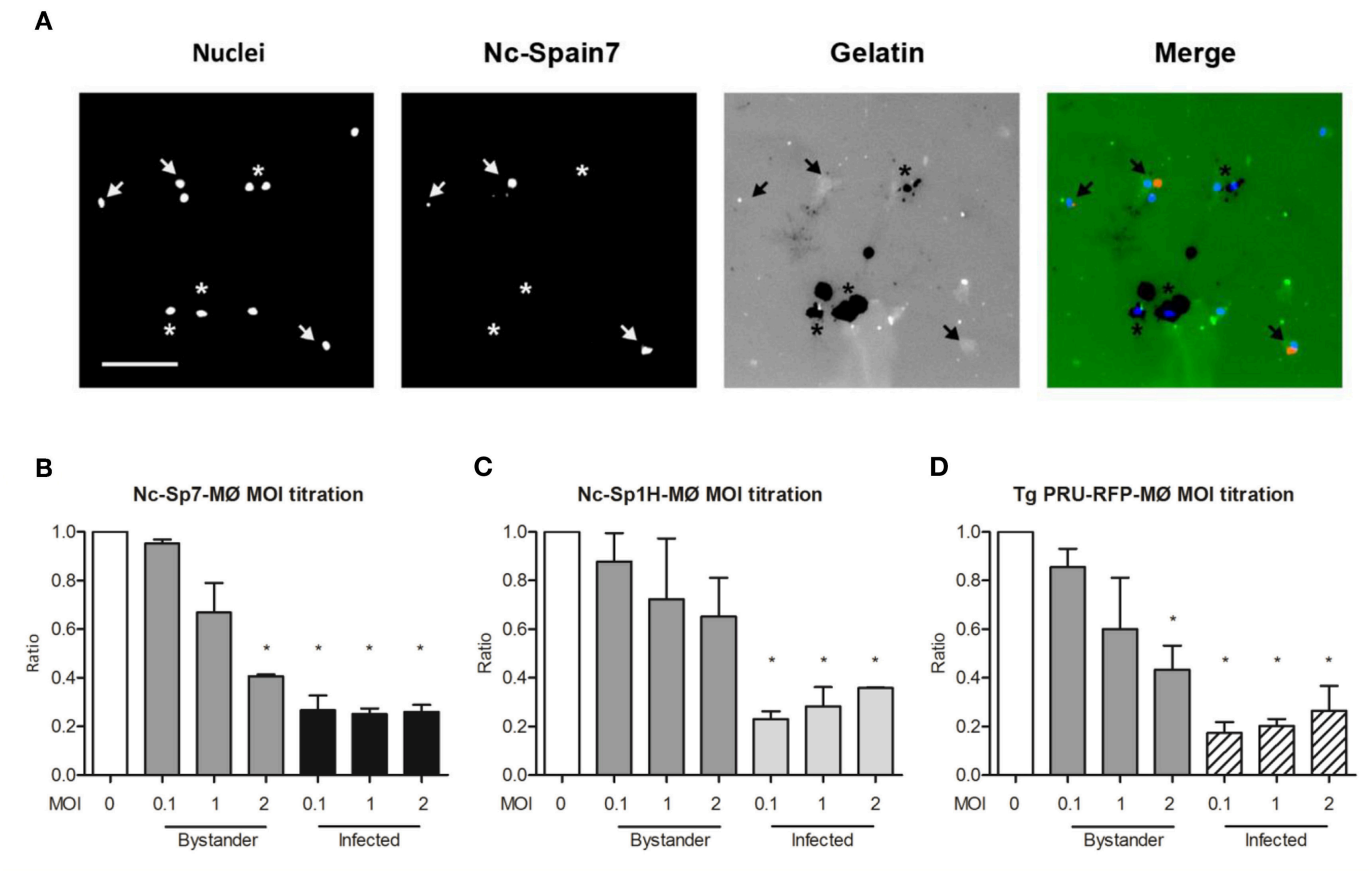
Pericellular proteolysis of bovine MØs upon challenge with *N. caninum* and *T. gondii*. Gelatin degradation of MØs challenged with *N. caninum* Nc-Spain7 (Nc-Spain7-MØ), Nc-Spain1H (Nc-Spain1H-MØ), or *T. gondii* PRU-RFP (*T. gondii* PRU-RFP-MØ) at the indicated MOIs. **(A)** Representative micrographs show MØ nuclear staining (Nuclei), Alexa-594-stained tachyzoites (Nc-Spain7), and fluorescent gelatin (Gelatin) with areas of gelatin degradation (absence of fluorescence signal). Arrowheads exemplify Nc-Spain7-infected MØs with an absence of gelatin degradation. Asterisks exemplify non-infected by-stander MØs and co-localization with gelatin degradation. Scale bar represents 100 μm. **(B–D)** Bar graphs show the mean (±SEM) relative gelatin degradation of unchallenged cells in CM (set to 1.0, MOI 0), non-infected by-stander cells (Bystander) and *Neospora* Spain7- **(B)**, Spain1H- **(C)** or *T. gondii*-infected **(D)** cells (Infected). The analysis comprised 160 FOVs and a total of 15.579 ± 1.087 (SEM) cells per condition from two independent experiments. Significant differences are indicated (*p < 0.05).

*Toxoplasma gondii* modulates the interaction of hypermigratory parasitized DCs with extracellular matrix (24, 25). To address if *N. caninum* infection had an impact on matrix degradation by boMØs, cells were challenged with *N. caninum* isolates, and the pericellular degradation of gelatin (denatured collagen) was assessed (Figure 4A). *T. gondii* PRU-RFP-challenged MØs were included in the assays as the positive control. The proteolytic activity of non-infected by-stander boMØs was also determined. A similar significant reduction in gelatin degradation was observed in boMØs infected with Nc-Spain7, Nc-Spain1H, and *T. gondii* PRU-RFP compared with unchallenged boMØs (*p* > 0.05). A dose-dependent (MOI) reduction in pericellular proteolysis was observed in by-stander MØs. In contrast, abrogation of the matrix proteolysis in infected boMØs was MOI-independent (Figures 4B–D).

We conclude that live intracellular *N. caninum* reduces or abrogates the matrix degradation capability of infected boMØs.

### Live *N. caninum* Tachyzoites Induce a Hypermigratory Phenotype in Bovine Macrophages

**Figure 5:**
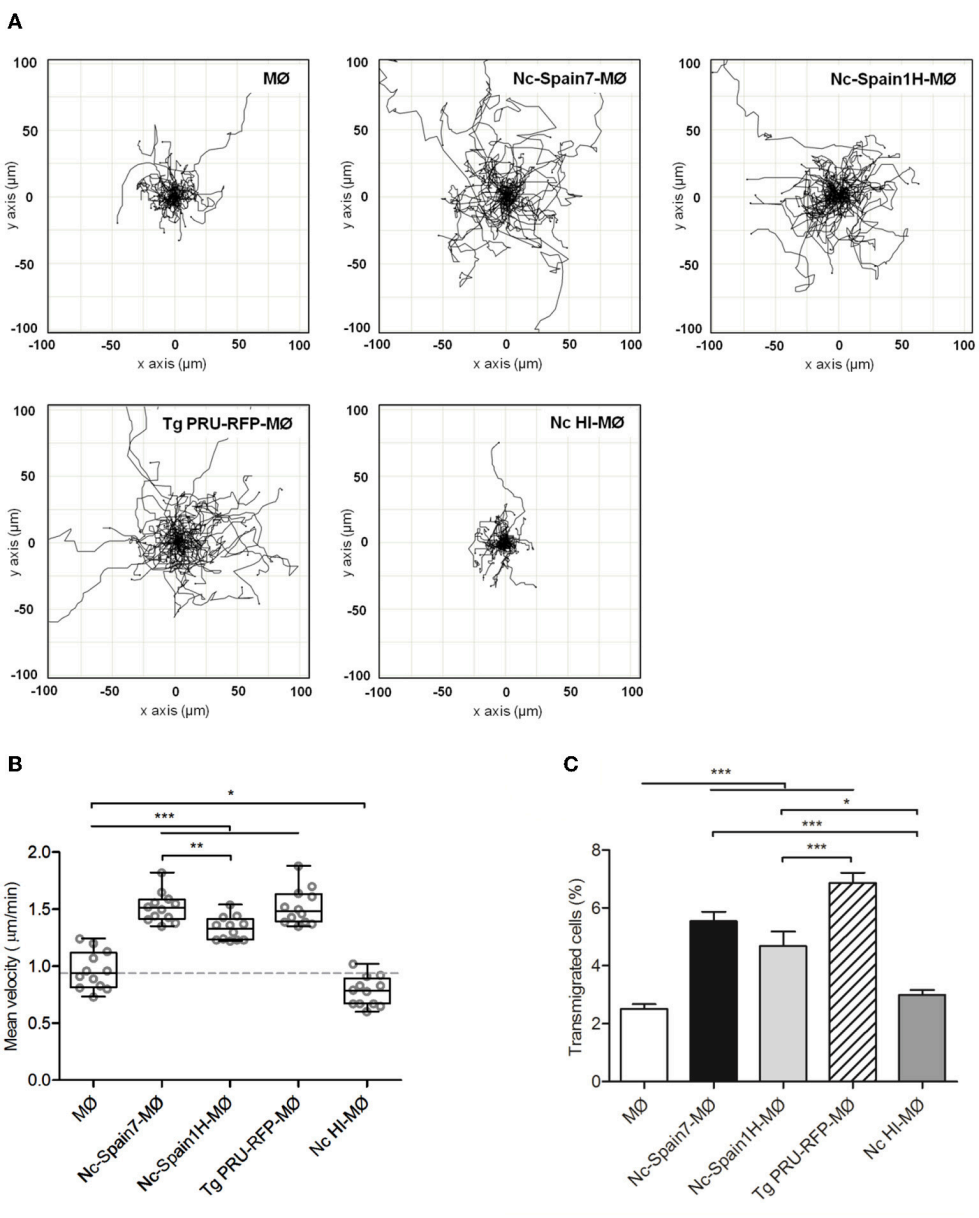
Hypermotile phenotype and enhanced transmigration in bovine MØs induced by *N. caninum* infection. **(A)** Motility plot of unchallenged MØs (MØ) and MØs challenged for 4 h with *N. caninum* Nc-Spain7 (Nc-Spain7-MØ), Nc-Spain1H (Nc-Spain1H-MØ), heat-inactivated tachyzoites (Nc HI-MØ) or *T. gondii* PRU-RFP tachyzoites (Tg PRU-RFP-MØ). **(B)** Graph represents the mean velocity and SEM of three independent experiments. Circles indicate the average velocity of 50 cells tracked in two different tiles and two different wells per experiment. Box plot shows upper and lower interquartile range with median. **(C)** The graph represents the percentage of transmigration 18 hpi of MØs, Nc-Spain7-MØs, Nc-Spain1H-MØs, Nc HI-MØs, and Tg PRU-RFP-MØs. The data represent the mean values and SD of three independent experiments; Significant differences are indicated (*p < 0.05; **p < 0.01; ***p < 0.001).

In murine *T. gondii* infections, induced hypermigration of parasitized DCs has been associated with enhanced parasite dissemination to peripheral organs (20, 22). To investigate whether *N. caninum* is able to exploit the migratory properties of boMØs, the motility and transmigration of boMØs challenged with Nc-Spain7 and Nc-Spain1H isolates were assessed. BoMØs challenged with the *T. gondii* PRU-RFP line were also included in the study. A significantly enhanced velocity was observed in boMØs challenged with both *N. caninum* and *T. gondii* parasites (*p* < 0.001). When comparing both *N. caninum* isolates, significantly higher elevated velocity values were recorded for the highly virulent isolate Nc-Spain7 (*p* < 0.01), which were similar to those for *T. gondii* (*p* > 0.05). In contrast, challenge of boMØs with *N. caninum* HI tachyzoites yielded velocities significantly lower than the baseline velocities of unchallenged boMØs (*p* < 0.05) (Figures 5A,B).

In transmigration assays, significantly higher transmigration frequencies were also recorded for boMØs infected with both *N. caninum* and *T. gondii* tachyzoites (*p* < 0.001) than for unchallenged boMØs. When comparing both *N. caninum* isolates, non-significant differences in transmigration frequency were found. *T. gondii* induced the highest enhanced transmigration, with statistically significant differences compared with Nc-Spain1H (*p* < 0.001) but not with Nc-Spain7 (*p* > 0.05). Challenge of boMØs with HI tachyzoites resulted in non-significantly altered transmigration frequency (*p* > 0.05) (Figure 5C).

Taken together, these results show for the first time that *N. caninum* induces a hypermigratory phenotype (hypermotility and enhanced transmigration) in bo MØs. A significantly higher hypermotility was observed for the highly virulent isolate Nc-Spain7 compared with Nc-Spain1H.

### The Highly Virulent Isolate Nc-Spain7 Completely Down-Regulates ROS Production in Infected Macrophages

**Figure 6:**
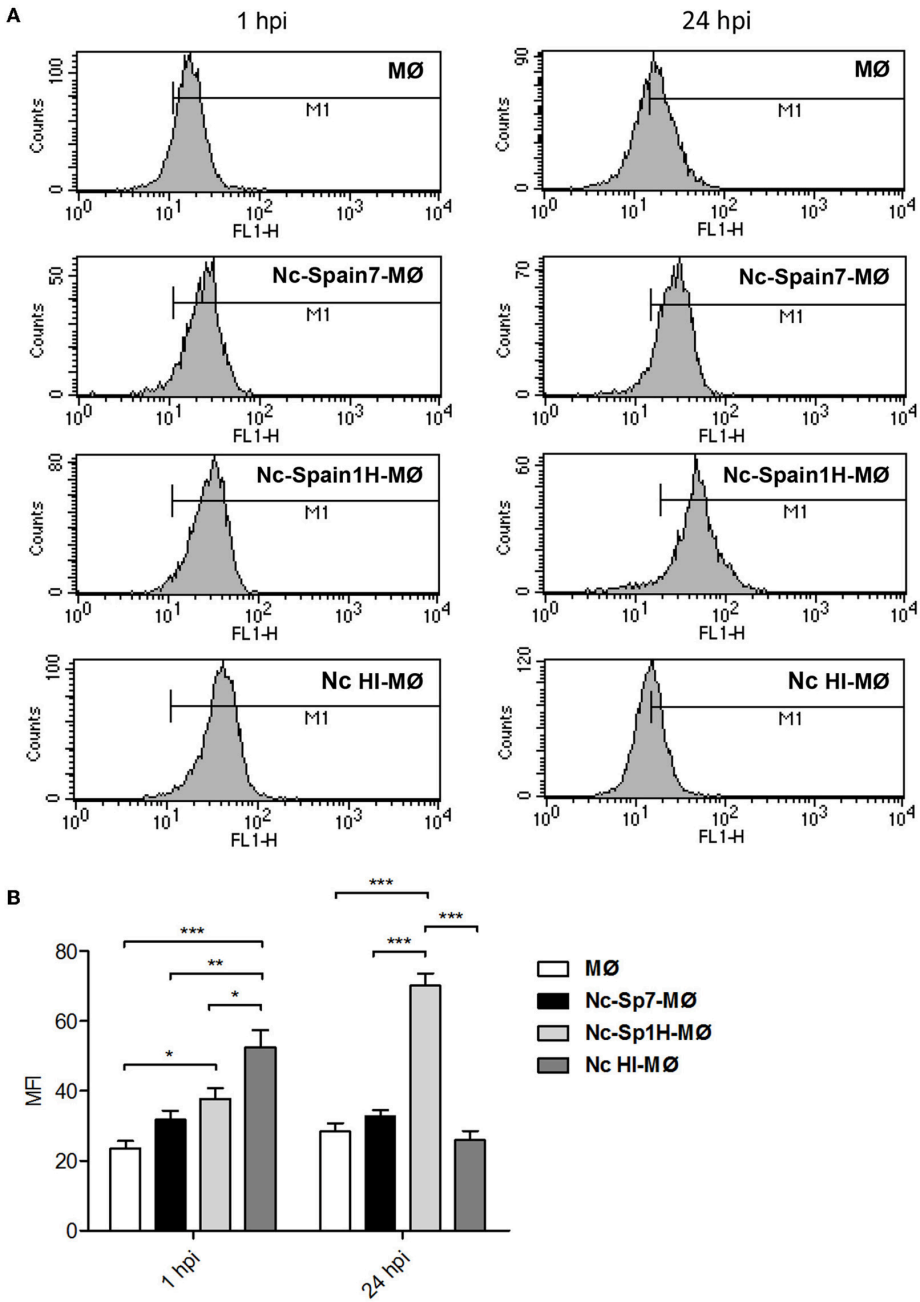
ROS generation induced by *N. caninum* infection. **(A)** Representative histograms show the mean fluorescence intensity (MFI) determined by flow cytometry analysis of non-infected MØs (MØ), MØs challenged for 1 and 24 h with *N. caninum* Nc-Spain7 (Nc-Spain7-MØ), Nc-Spain1H (Nc-Spain1H-MØ), and heat-inactivated tachyzoites (Nc HI-MØ) stained with CellROX Green Reagent. **(B)** The bar graph show the comparison of the MFI between groups. Each column and error bar represents the mean and SD of three replicates from three independent experiments; Significant differences are indicated (*p < 0.05; **p < 0.01; ***p < 0.001).

Having established the impact of different *N. caninum* isolates on boMØ morphology and cytoskeletal functionality, we next evaluated whether the infection also impact on innate immune parameters in a virulence-specific manner. To assess the ability of boMØ to kill invading *N. caninum* by production of ROS, differential ROS production upon *N. caninum* inoculation with Nc-Spain7, Nc-Spain1H, and HI tachyzoites was evaluated by flow cytometry at 1 and 24 hpi (Figure 6). At 1 hpi, a significant increase in ROS production was found in MØs inoculated with Nc-Spain1H (*p* < 0.05) and HI tachyzoites (*p* < 0.001) vs. non-infected cells but not in MØs inoculated with Nc-Spain7 (*p* > 0.05). However, at this time-point no significant differences were found between isolates, which both induced lower ROS generation by MØs compared with HI tachyzoites (*p* < 0.05). At 24 hpi, the production of ROS by Nc-Spain1H-infected MØs was enhanced (*p* < 0.001) compared to that of non-infected MØs as well as MØs incubated with HI tachyzoites. Interestingly however, no ROS production above either control or MØs incubated with HI tachyzoites was seen in MØs inoculated with the highly virulent isolate Nc-Spain7.

We conclude that live intracellular *N. caninum* reduces the ROS response of infected MØs at early infection, but only the highly virulent isolate Nc-Spain7 maintains the abrogated ROS production over time.

### *N. caninum* Infection of Macrophages Induces a Virulence-Dependent IL-10 and IL-12 mRNA Expression Pattern

**Figure 7:**
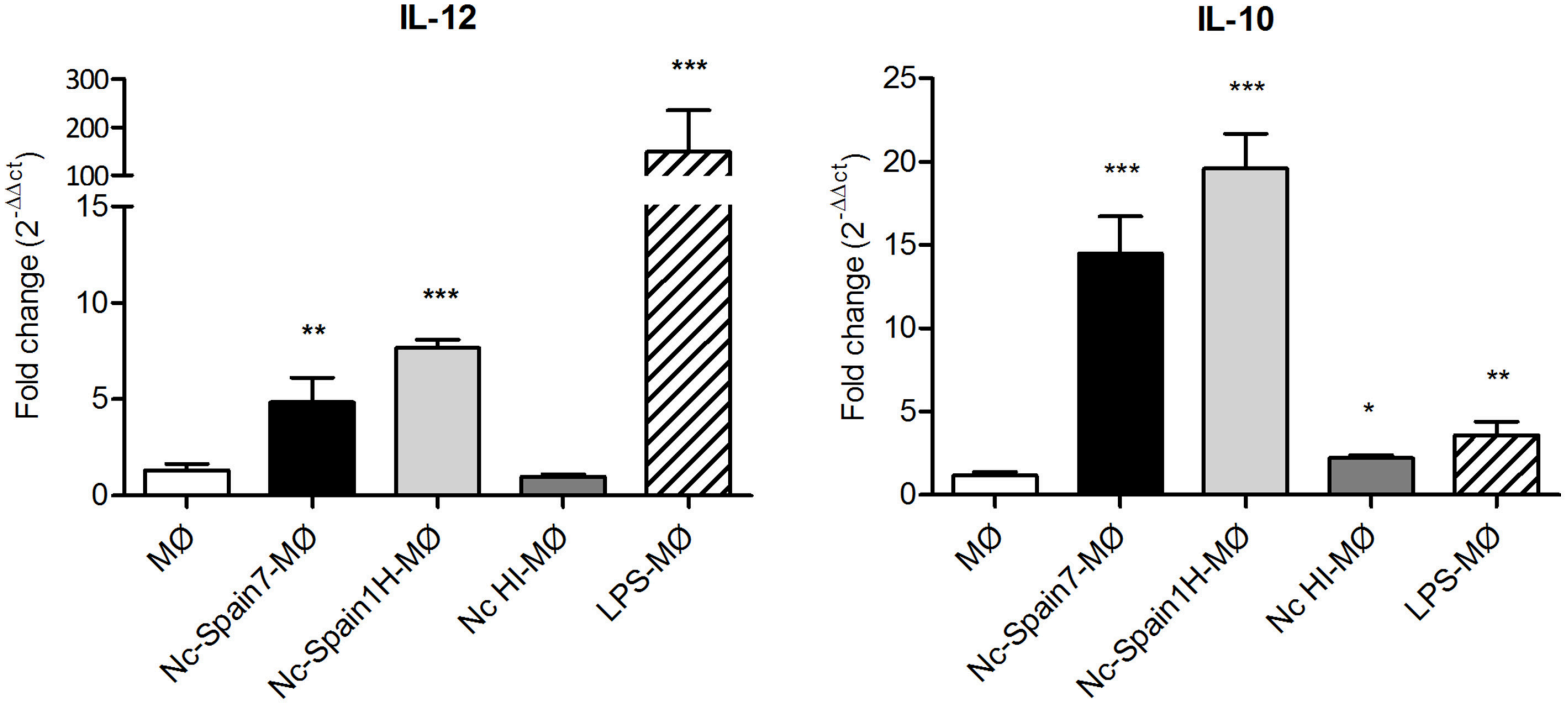
Cytokine mRNA expression in bovine MØs infected with *N. caninum*. Graphs represent the relative quantification of IL-10 and IL-12p40 mRNA expression levels (x-fold change in expression) in MØs challenged for 8 h with *N. caninum* Nc-Spain7 (Nc-Spain7-MØ), Nc-Spain1H (Nc-Spain1H-MØ), heat-inactivated tachyzoites (Nc HI-MØ), and stimulated with 100 ng ml^−1^ LPS (LPS-MØ) in relation to non-infected MØs (MØ). Significant differences are indicated (*p < 0.05; **p < 0.01; ***p < 0.001).

MØ have the ability to subsequently prime the adaptive immune response through secretion of cytokines, specifically the Th1-supporting IL-12, and the regulatory IL-10. Thus, in a next step, we investigated whether the differences in strain-specific ROS production would also be mirrored on the cytokine level. As expected, untreated MØ produced hardly any mRNA for either cytokine, whereas MØ incubated with LPS responded with a significantly enhanced mRNA expression for the pro-inflammatory cytokine IL-12 (148.7-fold; *p* < 0.001), and only mildly increased IL-10 mRNA production (3.6-fold; *p* < 0.01). Inoculation of MØ with HI tachyzoites did not increase IL-12p40 mRNA expression levels (0.99-fold; *p* > 0.05), and only marginally impacted on IL-10 mRNA expression (2.22-fold; *p* < 0.01) (Figure 7). In contrast, incubation of MØ with both types of live *N. caninum* tachyzoites induced a strain-specific increase in IL-12p40 mRNA and IL-10 mRNA expression compared to non-infected MØ, with the low virulent isolate Nc-Spain1H inducing more IL-12p40 (7.7-fold; *p* < 0.001) and IL-10 (19.6-fold; *p* < 0.001) than the highly virulent Nc-Spain7 isolate (4.8-fold; *p* < 0.01 and 14.5-fold; *p* < 0.001, respectively), although the differences between isolates were not statistically significant (*p* > 0.05).

In summary, live tachyzoites of both isolates induced IL-12p40 and IL-10 expression in boMØs. However, expression levels of both cytokines were higher for cells infected with the low virulent isolate Nc-Spain1H.

### *N. caninum* Infection Results in Changes in Surface Marker Expression in Bovine Macrophages

**Figure 8:**
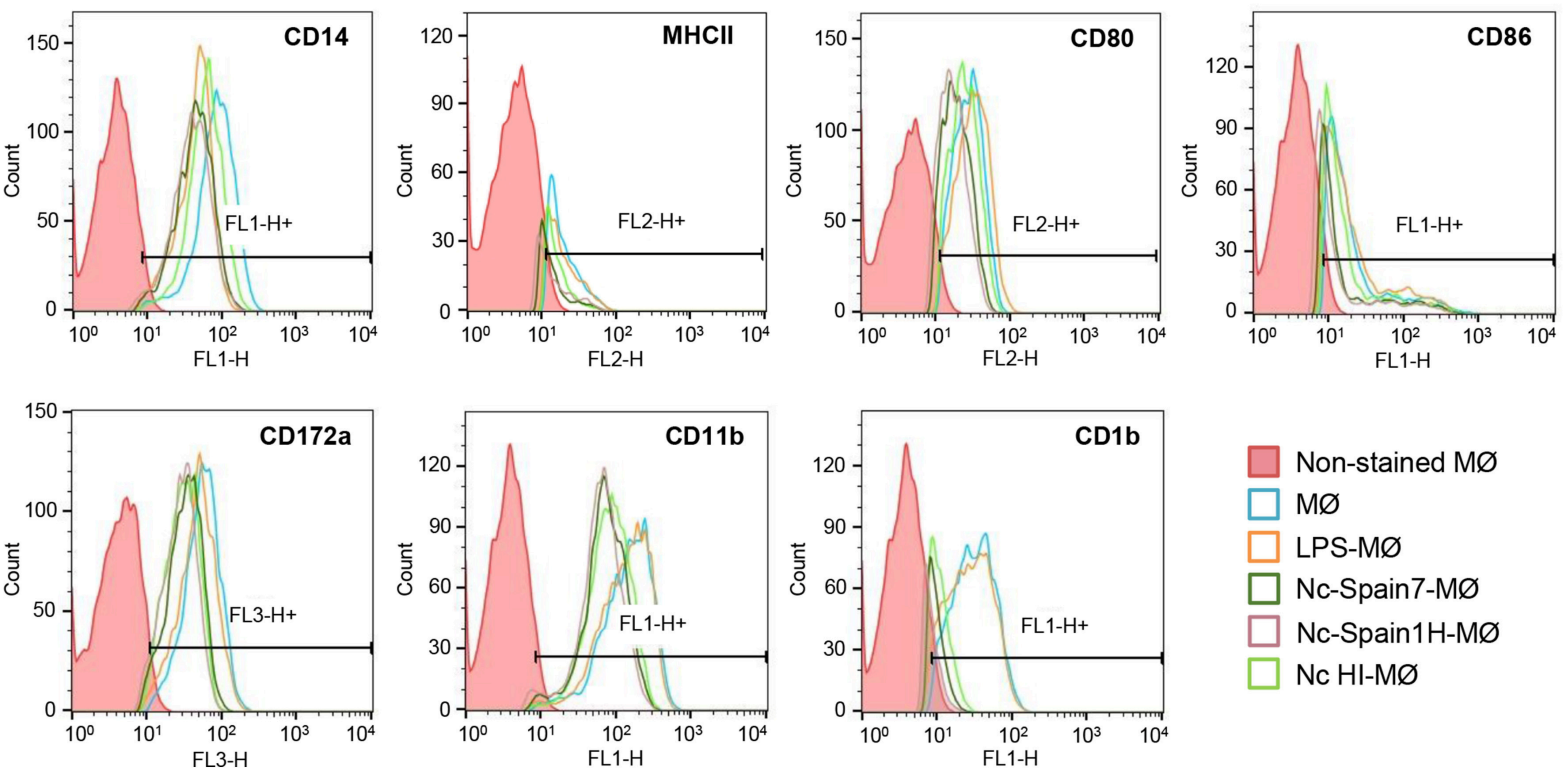
Phenotypic characterization of bovine MØs inoculated with Nc-Spain7, Nc-Spain1H, and HI tachyzoites. Histograms represent the percentage of positive cells and mean fluorescence intensity (MFI) of CD14, MHC class II, CD80, CD86, CD172a, CD11b, and CD1b markers determined by flow cytometry in non-infected MØs (MØ) and MØs challenged for 4 h with *N. caninum* Nc-Spain7 (Nc-Spain7-MØ), Nc-Spain1H (Nc-Spain1H-MØ), heat-inactivated tachyzoites (Nc HI-MØ), and stimulated with 100 ng ml^−1^ LPS (LPS-MØ). Histograms are representative of three independent experiments.

As the production of cytokines can also impact on surface antigen expression levels, we next analyzed the effects of the different *N. caninum* isolates on MØ activation by assessing surface antigen expression. Flow cytometry was used to quantify the expression of molecules involved in antigen presentation (CD1b, MHC Class II), T-cell activation and maturation (CD80, CD86), cell adhesion (CD11b), and phagocytosis (CD172a). As expected, MØ upregulated CD86 in response to LPS (*p* < 0.05). Infection of MØs with Nc-Spain7 or Nc-Spain1H resulted in a significant reduction in the percentage of MHC Class II (*p* < 0.05), CD86 (*p* < 0.05), and CD1b (*p* < 0.0001) expressing cells, whereas inoculation with HI tachyzoites resulted only in a significant reduction of MHC Class II (*p* < 0.05) and CD1b (*p* < 0.0001) (Table 1). The mean MFI of CD11b and CD1b diminished in MØs inoculated with Nc-Spain7, Nc-Spain1H and HI tachyzoites vs. non-infected MØs (*P* < 0.01–0.001; Figure 8). Significant differences between isolates were not found in the expression of any surface antigen studied.

**Table 1:** Immunophenotypic analysis by flow cytometry of bovine MØs exposed to *N. caninum*.

	**CD14**	**MHC** **class II**	**CD80**	**CD86**	**CD172a**	**CD11b**	**CD1b**
Monocytes	95.95 ± 4.52	85.40 ± 12.86	85.05 ± 2.47	61.45 ± 8.38	98.55 ± 0.21	98.90 ± 0.42	90.95 ± 1.25
MØ	95.2 ± 2.14	27.02 ± 10.89	89.20 ± 1.60	59.48 ± 7.78	96.47 ± 2.91	94.58 ± 3.41	93.33 ± 4.39
Nc-Spain7-MØ	95.57 ± 3.14	11.66^*^ ± 3.56	86.72 ± 5.13	47.40^*^ ± 14.99	94.87 ± 4.22	96.47 ± 3.48	43.07^*^ ± 13.44
Nc-Spain1H-MØ	95.17 ± 3.56	11.96^*^ ± 2.61	85.32 ± 5.15	43.30^*^ ± 13.60	94.05 ± 4.31	96.18 ± 3.60	31.42^*^ ± 8.11
Nc HI-MØ	95.95 ± 2.48	13.35^*^ ± 7.18	83.45 ± 3.41	54.46 ± 10.15	95.52 ± 3.19	97.07 ± 2.46	40.56^*^ ± 6.32
LPS-MØ	95.18 ± 3.13	21.01 ± 10.25	90.05 ± 4.91	70.81^*^ ± 9.60	96.00 ± 3.12	95.77 ± 2.23	92.92 ± 4.52

The results are expressed as the percentage of stained cells for each surface molecule in monocytes, non-infected MØs (MØ), and MØs challenged for 4 h with N. caninum Nc-Spain7 (Nc-Spain7-MØ) or Nc-Spain1H (Nc-Spain1H-MØ) isolates, heat-inactivated tachyzoites (Nc HI-MØ), and 100 ng ml^-1^ LPS (LPS-MØ). The data are presented as the mean values ± SD of three independent experiments. (*) Indicates significant differences in challenged MØs vs. non-infected cells (p < 0.05).

### Infection of Bovine Macrophages With the Highly Virulent Isolate Nc-Spain7 Resulted in Diminished IFN-γ Secretion by co-cultured Autologous Lymphocytes

**Figure 9:**
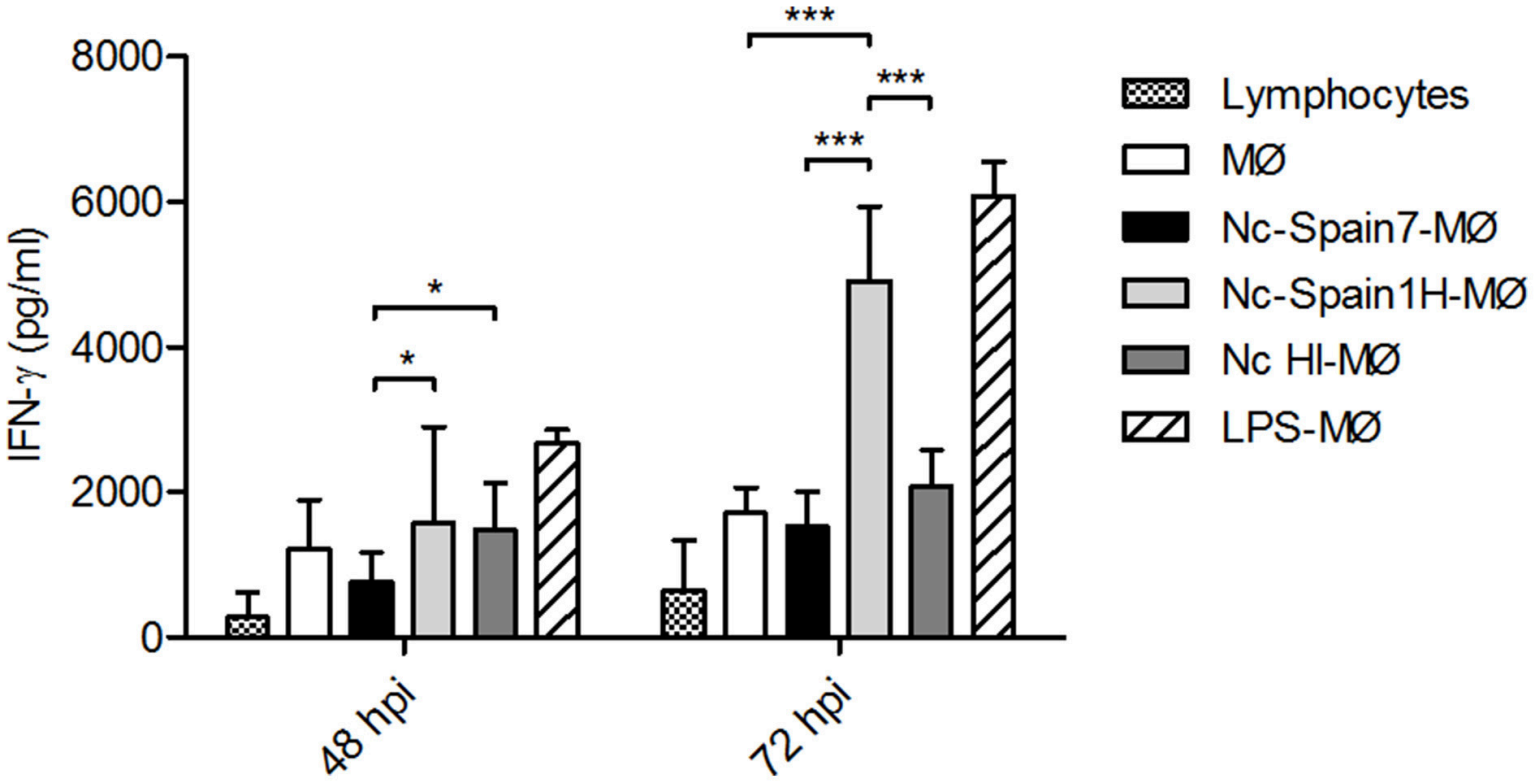
IFN-gamma production by lymphocytes in co-culture with *N. caninum*-infected MØs. The graph represents the concentration of IFN-γ in supernatants obtained from lymphocytes in co-culture with non-infected MØs (MØ) or MØs challenged with *N. caninum* Nc-Spain7 (Nc-Spain7-MØ), Nc-Spain1H (Nc-Spain1H-MØ), heat-inactivated tachyzoites (Nc HI-MØ) or 100 ng ml^−1^ LPS (LPS-MØ). Each column and error bar represents the mean and SD of three replicates from three independent experiments. Significant differences are indicated (*p < 0.05; ***p < 0.001). IFN-γ was not detected in samples without lymphocytes, and lymphocytes without macrophages (lymphocytes) reached average IFN-γ values lower than 1,000 pg ml^−1^.

As the previous data clearly indicated an isolate specific impact on the innate immune response created by boMØ to *N. caninum* isolates, we lastly assessed whether these differences would also affect the adaptive immune response. To do so, IFN-γ release by lymphocytes induced by *N. caninum*-infected MØs was assessed in the supernatant of co-cultures (Figure 9). As expected, the highest concentrations of IFN-γ were produced by lymphocytes when MØs were inoculated with LPS (*p* < 0.0001). MØs infected with Nc-Spain1H also stimulated IFN-γ production by lymphocytes, higher production than that by MØs infected with Nc-Spain7, at 48 hpi (*p* < 0.05) and 72 hpi (*p* < 0.0001). Exposure to Nc-Spain7 or HI tachyzoites did not result in significant IFN-γ variations compared with the negative control (co-culture of non-infected MØs and lymphocytes) at any time point. Samples of lymphocyte cultures reached average values of IFN-γ lower than 1,000 pg ml^−1^, and IFN-γ was not detected in MØ cultures without lymphocytes (MØs, MØs inoculated with Nc-Spain7, Nc-Spain1H, HI tachyzoites or LPS).

Altogether, our results showed that the IFN-γ response was impaired by Nc-Spain7 infection.

## Discussion

*N. caninum* virulence is likely determined by several factors, such as invasiveness, replication capability, and the strength and characteristics of the induced immune response in the host. However, specific virulence factors for *N. caninum* in cattle have not yet been determined and the mechanisms used by the parasite to modulate bovine host cells during the early phase of infection are still poorly understood, supporting the need for new investigations. Recently, large-scale transcriptomic and proteomic studies carried out by our group in a bovine trophoblast cell model highlighted a very similar regulation pattern by Nc-Spain7 and Nc-Spain1H isolates, even though the low virulence isolate seems to exert a higher modulation of the host cell (38). Additionally, a different expression of genes involved in invasion, metabolic processes, cell cycle, and stress response were described for Nc-Spain7 and Nc-Spain1H (38, 39).

Studies in murine and human *in vitro* cellular models suggest that infection of antigen presenting cells (MØs and DCs) by *N. caninum* may have modulatory effects on cytokine secretion and lymphocyte activation (4, 7, 10, 40). However, mice and humans are not natural hosts for *N. caninum*, so these models may not accurately reflect the dynamic of the host (bovine)–pathogen relationship. Thus, the ability of the parasite to initiate innate immune responses should be determined in the bovine host.

BoMØs can be used as an infection model for identification of strain-specific virulence properties or mechanisms to subvert immune response activation. Here, we studied for the first time the interaction between boMØs and two *N. caninum* isolates of different virulence (14, 16, 28).

The *N. caninum* lytic cycle in host cells comprises the processes of invasion, adaptation to intracellular conditions, proliferation, and egress from host cells. This sequence of events is required for parasite survival and propagation in the course of infection. The ability of *N. caninum* to infect, survive, and replicate in boMØs was studied. A comparison of infection rates showed that both isolates were internalized into boMØs with a higher efficiency than described for other cell lines (13, 14, 30). This fact can be due because MØs are phagocytic cells, which would enable tachyzoites to enter MØs by two complementary routes: uptake by MØs and active invasion by the parasite. In a recent study in human MØs, the internalization of *N. caninum* was found to likely occur via active invasion (10). In our study both processes were involved in the internalization of *N. caninum* tachyzoites, with differences found between isolates in the percentages attributable to active invasion, similar to those determined in previous studies (13, 30). Different receptors and signaling pathways could be implicated depending on how the parasite is internalized. These might facilitate replication of the parasite in the parasitophorous vacuoles when active invasion occurs or killing and degradation of the parasite if tachyzoites are internalized via endocytosis.

When parasite proliferation was monitored over time, *N. caninum* replication efficacy in boMØs was lower than in other types of cells previously assayed (13, 30), suggesting the ability of MØs to limit the growth of the parasite. Furthermore, the efficient growth of Nc-Spain7 in boMØs, significantly higher than that of Nc-Spain1H, suggests the success of the highly virulent isolate in establishing an intracellular replicative niche in boMØs. The higher growth rate of Nc-Spain7 could be related to more efficient mechanisms to obtain energy and multiply intracellularly (38, 41) and to the ability of the isolate to modulate the immune response, which would allow the parasite to survive and replicate more efficiently in MØs.

Another phenotypic trait putatively related to virulence is the capacity to modulate the migratory properties of parasitized immune cells, which may potentiate parasite dissemination in the host passage to immunoprivileged sites (i.e., brain, placenta) by crossing biological barriers, transmission, or establishment of chronic infection. Previous studies have described the induction of a hypermigratory phenotype in *T. gondii*-infected murine and human MØs and DCs characterized by hypermotility (21), enhanced transmigration across cellular barriers (20, 37), and extracellular matrix, and rapidly induced cytoskeletal changes post-invasion (23). These processes are consistent with amoeboid migratory activation of the infected leukocyte (24). In the present work, we report for the first time the induction of hypermigration by *N. caninum* in boMØs. Additionally, we describe the ability of *T. gondii* to induce hypermigration in boMØs, previously described in human and murine MØs (42). Moreover, shortly after infection with *N. caninum*, boMØs exhibited dramatic morphological changes, and abrogated pericellular proteolysis, all consistent with non-proteolytic amoeboid migratory activation in boMØs and in line with effects reported for *T. gondii* in murine and human DCs (23, 24). In line with the above, podosome dissolution was observed in *Neospora*-infected boMØs. Podosomes are important cytoskeletetal structures linked to cell signaling, adhesion, matrix degradation, and therefore migration. The dissolution of podosomes upon infection by *N. caninum* implies fundamental mechanistic changes in how the parasitized cells interact with the surrounding microenvironment and migrate. Specifically, integrin-mediated adhesion and signaling and metalloproteinase activity is focalized and regulated in podosomes (24, 25). Because parasite-induced leukocyte hypermigration has been linked with enhanced dissemination for *T. gondii* in mice (20) and in a murine neosporosis infection model (31), this motivates further studies to address its putative impact in bovine *Neospora* infection. Additionally, how *N. caninum* molecularly orchestrates the migratory activation of parasitized boMØs and the identification of implicated parasite and host cell-mediated signaling, awaits further investigation. The elucidation of these processes may provide novel insights in how *N. caninum* evades immune responses while ensuring dissemination, and ought to be relevant for vaccination strategies in bovines.

Jointly, the data demonstrate that boMØs respond with a migratory phenotype upon infection with *N. caninum* and *T. gondii*. Contrary to *N. caninum, T. gondii* is not a prominent pathogen in bovines. However, the data are consistent with the existence of a conserved mechanism between *N. caninum* and *T. gondii* for migratory activation of infected leukocytes. Consequently, the utilization of a Trojan horse mechanism for systemic dissemination (22) may therefore be a conserved strategy for these two coccidian parasites (31). Despite that virulence traits are complex and can be host species-specific (43), our findings also indicate that both virulent and non-virulent *N. caninum* isolates have the potential to use boMØs to disseminate and suggest that the virulent isolate Nc-Spain7 may have a superior capacity to induce migration of boMØs. However, further studies with a larger panel of isolates are necessary to determine if variations in the induction of hypermigration could be related to differences in transmission and dissemination found *in vivo* (31). Altogether, this highlights that (*i*) targeted survival in MØ/DCs constitute a replicative niche and that (*ii*) interference with migration of parasitized MØ/DCs are conserved features for the two coccidia, as shown with cells of bovine, murine and human origin. It also advocates for evolutionary conserved dissemination strategies by coccidia in vertebrates.

An efficient cell-mediated Th1 immune response is critical for restricting parasite replication (44, 45). The expression of IL-12 by MØs stimulates lymphocytes to produce IFN-γ, which is important for restricting intracellular replication due to its role in activating MØ-mediated mechanisms that kill intracellular pathogens and T-cell proliferation (46). ROS generation is also an important mechanism in the control of *N. caninum* replication (9). Persistent intracellular parasites such as *T. gondii* or *Leishmania* are able to modulate mitochondrial and NADPH oxidase-induced ROS with the aim to maintain long-term carriage by avoiding their clearance (47–49). Mechanisms of modulation of intracellular ROS by infection of intracellular pathogens include interference of NADPH complex assembly; scavenging of ROS produced by NADPH oxidase and interference of mitochondrion-based ROS production during infection (48). In the present study, infection of boMØs with viable *N. caninum* tachyzoites repressed ROS, and IFN-γ production at the early stages of infection, which would result in an inability to control the infection by the host. Moreover, our results also indicate that Nc-Spain7 shows a higher ability to evade the host immune response, since lower ROS and IFN-γ production, as well as lower IL12p40 expression, was observed. In a previous work, we showed that the glucose-6-phosphate dehydrogenase (G6PD) was more abundantly expressed in Nc-Spain7 than Nc-Spain1H tachyzoites (39). This enzyme is indispensable to the maintenance of the cellular redox balance and detoxification of ROS by producing NADPH (50). The level of G6PD activity of the isolates may determine their sensitivity to oxidative stress and thus its ability to reduce ROS levels and facilitate the survival into MØs. Further studies are necessary to determine the role of *N. caninum* G6PD in bovine MØs.

In contrast, the less virulent isolate Nc-Spain1H elicits a stronger Th1 response initiated by boMØs, which would control parasite replication and dissemination in the host. In this sense, highly immunogenic cell surface proteins are differentially regulated between isolates, being more abundant on the Nc-Spain1H isolate (38, 41), which may explain the stronger stimulation of the immune system. In previous studies we described that infection by the low-virulence isolate Nc-Spain1H induced a higher expression of TLR-2, starting an inflammatory response in bovine trophoblast cells, which may be the cause of the lower proliferation of this isolate (38, 51). TLR activation is crucial for initiating the innate immune responses responsible for the elimination of intracellular parasites such as *N. caninum*. The signaling pathway activated by TLR-2 leads to an increase in the transcription factors NF-κβ and AP-1, which trigger the synthesis of pro-inflammatory cytokines (52). The implication of TLR-2 in *N. caninum* recognition has been also described in murine bone marrow derived MØs, activating proinflammatory signaling pathways which leads to production of Th-1 cytokines including IL-12 (53, 54). In *T. gondii* infections, strain-specific differences in NF-κB signaling were observed in murine bone marrow-derived macrophages, with higher levels of NF-κB activation and IL-12 production induced by type II parasites (considered low virulent in mice) than type I strains (high virulent in mice) (12).

On the other hand, regulatory cytokines, such as IL-10, are required to limit the response against *N. caninum*, protecting the host from infection-associated immunopathology (46, 55). High IL-10 expression levels by *N. caninum*-infected MØs were observed for both isolates (being higher for Nc-Spain1H) but not in MØs inoculated with HI tachyzoites, suggesting a modulatory role of live *Neospora* in the immune response generated during infection to guarantee host survival. As it has been suggested for *T. gondii*, succeeding in finding a balance between pro- and anti-inflammatory responses may be a possible strategy to establish a chronic infection, promoting survival of both parasite and host (18). Thus, the higher levels of IL-12 induced by the low virulent isolate may justify its higher expression levels of IL-10 in an isolate that is able to transmit without causing pathology (16).

Modulation of cell surface proteins was also induced by *N. caninum* infection. Diminished MHC Class II, CD1b, and CD86 presentation was observed on the surface of infected MØs at 4 hpi, which could be related to parasite immune evasion strategies. The reduction in MHC Class II has also been observed in *T. gondii-*infected murine MØs (56, 57), where the interference with the antigen presentation pathway has been indicated as an important strategy for intracellular survival of the parasite. A similar strategy may be assumed for *N. caninum*, supported by the fact that up-regulation of MHC class II on *N. caninum* murine MØs has been associated with host survival (58). In addition, lower CD1b expression has been correlated with a reduced induction of specific T-lymphocyte proliferation (59). The decrease in CD86 observed in MØs inoculated with live but not HI tachyzoites could suggest a modulation induced by components secreted by actively replicating parasites. CD86 is a co-stimulatory signal for activation of lymphocytes (60). A reduction in CD86 expression has also been observed in *Leishmania-*infected MØs (61) and in murine peritoneal MØs exposed to *N. caninum* (7). These data could indicate that *N. caninum* would interfere with intracellular signaling to selectively reduce antigen presentation and the expression of ligands related to T-cell activation, a mechanism to escape the host immune response. In the current study, no major differences in the expression of the different surface markers between isolates were observed at 4 hpi, being necessary more studies at different time points. Moreover, the functional consequences of the infection by different parasite isolates in boMØs should be investigated in future studies.

In summary, the results of the present study indicate that the highly virulent isolate Nc-Spain7 shows a higher ability to evade the host immune response via a reduction in ROS and IFN-γ secretion, which could be attributable to a lower IL-12 expression. The consequence would be lower control of intracellular parasite survival, which in conjunction with its higher replication and transmigration capacity may be associated with the higher virulence and pathology found *in vivo*. On the other hand, Nc-Spain1H elicits a strong Th1 response (which would reduce the parasite load in host cells) and that would be counterbalanced by a higher expression of regulatory cytokines (IL-10), minimizing pathology. This strategy may suggest a better adaptation of the low virulence isolate Nc-Spain1H to be transmitted without causing clinical disease, maintaining a delicate balance between suppression, and induction of the host immune response to ensure host survival and chronic infection. Our results also suggest that boMØs may serve as a vehicle for *N. caninum* dissemination throughout the organism to find the optimal host cells for replication (e.g., epithelial cells in the placenta) and as a niche for parasite long-term survival.

Understanding how *N. caninum* manipulates inhibitory signaling affords promising opportunities to counteract the parasite escape strategies and tip the balance in favor of the host. Further in-depth studies are needed to study the specific pathways modulated by *N. caninum* and to examine the differences between high and low virulence isolates that may explain the differences observed in their biological behavior. Proteomic approaches based on the study of differential protein determinants between Nc-Spain7 and Nc-Spain1H may help explain differences in the strength and character of the immune response induced by the isolates. In addition, experimental *in vivo* infections in cattle are necessary to determine if the results obtained in our *in vitro* study correspond with an expected enhanced early protective response to infection induced for the less virulent isolate Nc-Spain1H.

## Ethics Statement

This study was carried out in accordance with Spanish and EU legislation (Law 32/2007, Royal Decree 53/2013, Directive 2010/63/UE). The protocol was approved by the Ethical Committee of the Council of Agriculture, Farming and Autoctonous Resources of the Principality of Asturias, Spain (permit number PROAE 25/2016) and the Animal Welfare Committee of the Community of Madrid, Spain (permit number PROEX 236/17).

## Author Contributions

LO-M, JR-C, and EC-F conceived the study and PH, AKB, AB, and DW participated in its design. MG-S, LJ-P, and EC-F wrote the manuscript, with interpretation of results, and discussion input from PH, JR-C, AKB, AB, DW, and LO-M. MG-S and LJ-P performed all the experiments described in the manuscript. EÓ performed the gelatin degradation assay. JR-C and PH designed the RT-qPCR analyses, MG-S and LJ-P carried out the statistical analyses and interpreted the results. All authors read and approved the final manuscript.

### Conflict of Interest Statement

The authors declare that the research was conducted in the absence of any commercial or financial relationships that could be construed as a potential conflict of interest.
