# Supplementary material for: Differential Responses of Bovine Monocyte-Derived Macrophages to Infection by Neospora caninum Isolates of High and Low Virulence
Source: Front Immunol. 2019 Apr 30;10:915. doi: 10.3389/fimmu.2019.00915 (PMC6503000; doi:10.3389/fimmu.2019.00915)
Supplement: Supplementary file 1 [file Table_1.docx]

**Supplementary Table 1. List of antibodies used for immunophenotypic analysis of bovine monocyte-derived macrophages and characterization of lymphocytes.**

| **Antibody** | **Conjugate** | **Supplier** | **Species** | **Type** | **Clone** | **Reference** |
| --- | --- | --- | --- | --- | --- | --- |
| **CD14** | FITC | Bio-Rad Laboratories (Pleasanton, CA, USA) | Mouse anti-bovine | Monoclonal | CC-G33 | MCA2678F |
| **MHC Class II** | RPE |  |  |  | CC108 | MCA5656PE |
| **CD80** | RPE |  |  |  | IL-A159 | MCA2436PE |
| **CD86** | FITC |  |  |  | IL-A190 | MCA2437F |
| **CD172a** | RPE-Cy5 |  |  |  | CC149 | MCA2041C |
| **CD11b** | FITC |  |  |  | CC126 | MCA1425F |
| **CD1b** | Alexa Fluor  488 | Novus Biologicals  (Littleton, CO, USA) |  |  | CC20 | NB100-65315AF488 |
| **CD4** | Alexa Fluor  647 | Bio-Rad Laboratories (Pleasanton, CA, USA) |  |  | CC8 | MCA1653A647 |
| **CD8** | RPE |  |  |  | CC63 | MCA837PE |
| **CD335** | Alexa Fluor  488 |  |  |  | AKS1 | MCA2365A488 |
| **WC1** | FITC |  |  |  | CC15 | MCA838F |
| **CD21** | FITC |  | Mouse anti-human |  | LB21 | MCA1195F |
