# Supplementary figures and images for: Differential Responses of Bovine Monocyte-Derived Macrophages to Infection by Neospora caninum Isolates of High and Low Virulence
Source: Front Immunol. 2019 Apr 30;10:915. doi: 10.3389/fimmu.2019.00915 (PMC6503000; doi:10.3389/fimmu.2019.00915)

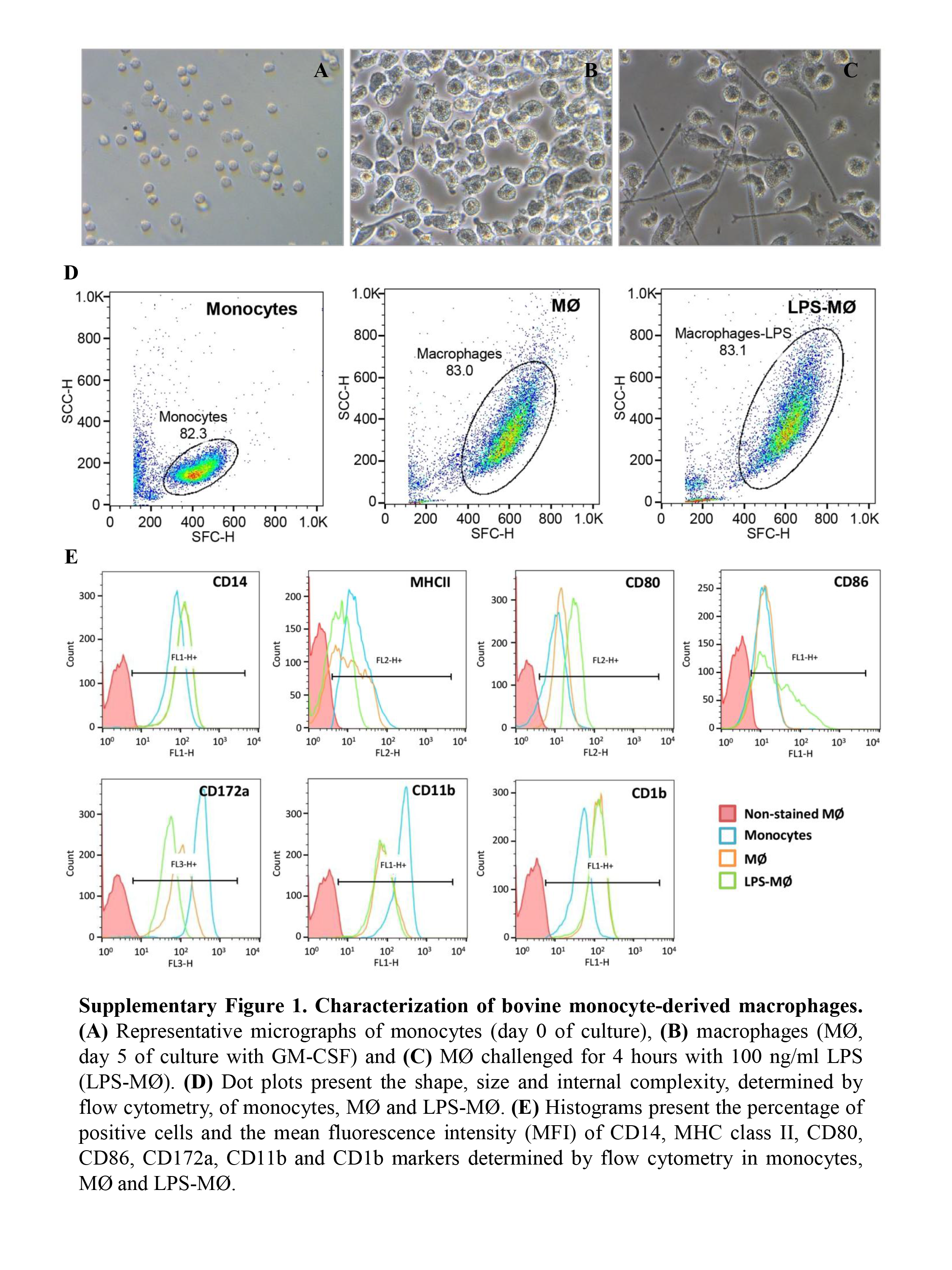

Supplement: Supplementary file 2 [file Image_1.TIF]

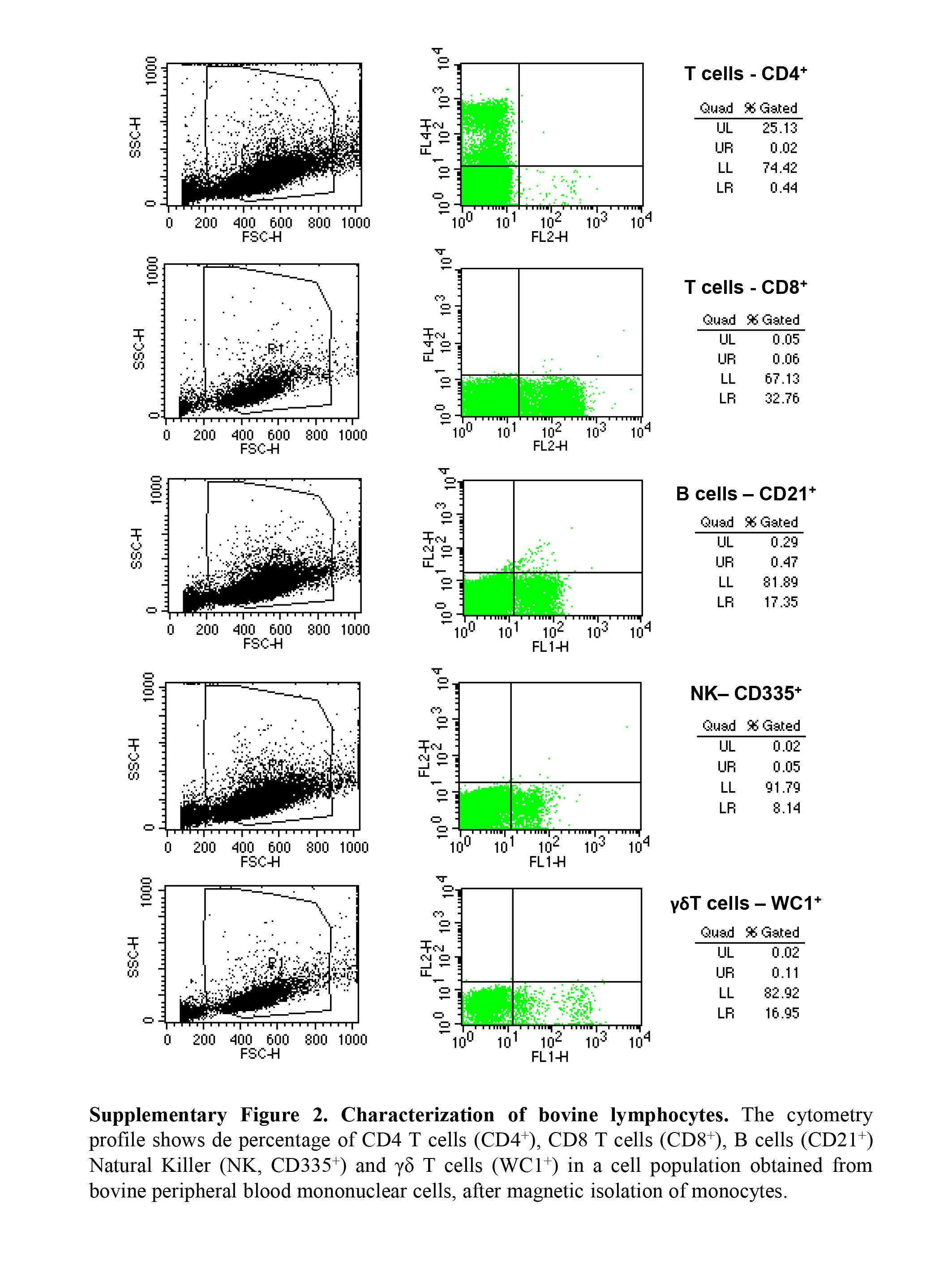

Supplement: Supplementary file 3 [file Image_2.TIF]
